# Managing infectious aerosols to counter engineered pandemics: Current recommendations and future research

**DOI:** 10.1111/risa.70054

**Published:** 2025-07-07

**Authors:** Adam Lerner, Gediminas Mainelis, William Hallman, Howard Kipen, Monica Magalhaes, Brian Buckley, José Guillermo Cedeño Laurent, Nir Eyal

**Affiliations:** ^1^ Institute for Health, Health Care Policy and Aging Research, School of Public Health Rutgers University New Brunswick New Jersey USA; ^2^ Department of Environmental Sciences, School of Environmental and Biological Sciences Rutgers University New Brunswick New Jersey USA; ^3^ Environmental and Occupational Health Sciences Institute, School of Public Health Rutgers University Piscataway New Jersey USA; ^4^ Department of Human Ecology School of Environmental and Biological Sciences, Rutgers University New Brunswick New Jersey USA; ^5^ Fundação José Luiz Egydio Setúbal São Paulo São Paulo Brazil; ^6^ Department of Philosophy School of Arts and Sciences Rutgers University New Brunswick New Jersey USA

**Keywords:** airborne disease transmission, ethics, health communication, indoor air quality, SARS‐CoV‐2, ventilation

## Abstract

In the increasingly likely event of an engineered‐virus outbreak or pandemic of catastrophic potential, managing infectious aerosols to reduce transmission will be crucial. Now is the time to start preparing our buildings, public opinion, and regulatory environments for the infectious aerosol management interventions necessary to protect the public. But which interventions should governments and institutions invest in the most? We review the leading candidate methods for infectious aerosol management and discuss their respective advantages, disadvantages, and suitable settings. There is strong emerging evidence that two recently explored technologies, direct exposure to far‐ultraviolet‐C (UVC) light and triethylene glycol, are particularly efficacious and safe, but there remain open questions about the long‐term safety and efficacy of these interventions. In the meantime, we recommend other interventions—especially upper‐room UVC and in‐room air cleaners—for settings where most occupants regularly spend more than a small fraction of their day. We conclude by listing research questions about these interventions that still need to be researched in social science, product development, medicine, engineering, economics, and ethics.

## 1 A DEVELOPING GLOBAL HEALTH CHALLENGE, WITH DEVELOPING SOLUTIONS

Advances in gene editing and synthesis enhance our ability for rapid and increasingly cheap genome engineering. These capabilities will advance human health in innumerable ways. But they could also be extremely dangerous. Research on the genetic features of pathogens that make them more communicable and lethal is progressing rapidly, aided by artificial intelligence (Adamala et al., 2024; Bobier et al., 2025; Caruso, 2023; Drexel & Withers, 2024; Dybul, 2023; Walsh, 2025). At the same time, individuals can already combine long DNA fragments purchased online, and affordable benchtop DNA printing may soon allow individuals to create biological entities or parts from scratch (Carter & Yassif, 2023). Together, this ongoing research and increasing access to technology could within years endow tens of thousands of individuals around the world (Esvelt, 2022) with the capacity to create especially dangerous pathogens—including viruses that are more lethal, communicable, infectious pre‐symptomatically, and evasive of vaccines than, for example, any SARS‐CoV‐2 variant (Esvelt, 2022; Jansen et al., 2014). Omnicidal individuals, terrorist organizations, rogue states, and even states that develop such dangerous pathogens for biodefense may intentionally or accidentally release these engineered pathogens (Paxton et al., 2024; Taylor, 2023). Such outbreaks might then result in pandemics significantly worse than any on record (Adamala et al., 2024; Esvelt, 2022; Gopal et al., 2023; Ord, 2020). Containing these outbreaks and potential pandemics requires effective national and global public health responses.

The armamentarium of responses to outbreaks of human‐engineered pathogens and other highly transmissible pathogens includes various pharmaceutical interventions (e.g., vaccines, antiviral medications) and non‐pharmaceutical interventions (e.g., masks, social distancing). In response to human‐engineered pathogens, management of indoor air (e.g., removal of airborne virus‐carrying particles or inactivation of viruses) is especially important. First, the most communicable forms of infectious diseases tend to be airborne and are small and aerosol‐mediated rather than sexually, large‐droplet, fomite, or vector‐mediated. Consequently, proper management of air indoors, where we spend 90% of our time, is especially pertinent to reducing the spread of these disaease. Second, weaponized pathogens are likely to be especially deadly, communicable, infectious pre‐symptomatically, and evasive of likely vaccines; it is therefore especially important to reduce exposures to them as soon and as much as possible. Third, like most other non‐pharmaceutical interventions, when successful, indoor aerosol interventions provide relatively broad‐spectrum protection. That is critical, because we do not know in advance what pathogen might be engineered, yet broad‐spectrum protections can be rolled out preemptively. This broad‐spectrum nature has the important further advantage of not tipping malevolent actors as to what we consider to be the worst threats. Finally, indoor‐air management can be active even before we detect an outbreak, which can help thwart pathogens that spread pre‐symptomatically. Alternative strategies (e.g., masking, isolation) focus on source control (i.e., on preventing pathogen release into shared indoor air) and, in principle, could also slow the spread of such pathogens preemptively. But their preemptive deployment could result in widespread noncompliance and in economic, educational, and mental health deficits. Other source control techniques (e.g., testing, air monitoring, and surveillance) may avoid these problems, but most could be used preemptively only to limit the spread of known pathogens, not unknown engineered pathogens. Some indoor‐air control systems may operate in the background without attracting attention.

Assuming that we must address the threat of human‐engineered pathogens, that doing so requires managing indoor air, and that we have limited resources, it follows that we must set priorities between different strategies for managing indoor air. The main strategies for containing outbreaks by managing indoor air include

(1) diluting air with outdoor air via natural or mechanical ventilation;
(2) filter pathogens out of air via
(2a) in‐duct filtration systems,
(2b) commercially available portable air cleaners (PACs), or
(2c) do‐it‐yourself (DIY) air cleaners (e.g., Corsi‐Rosenthal boxes),
and
(3) killing or inactivating pathogens by
(3a) irradiating air passing through air ducts with germicidal ultraviolet‐C (UVC) lighting,
(3b) irradiating air passing through upper, unoccupied parts of the room with germicidal UVC lighting
(3c) irradiating air in both unoccupied and occupied parts of rooms with direct far‐UVC lighting, or
(3d) spraying air sanitizers, especially recently proposed triethylene glycol (TEG)‐based systems, into rooms both when occupied and unoccupied

Deciding between these strategies requires making trade‐offs between, for example, their effectiveness, safety, cost, convenience, comfort (e.g., quietness), and public acceptability, which are intricate, interrelated, and not always clear‐cut. This review adds to the existing body of literature by explaining how to make these trade‐offs when selecting strategies to mitigate the threat of engineered pandemics. We describe the key criteria for choosing infectious aerosol management interventions to mitigate engineered pandemics (section II), use these criteria to highlight the key advantages and disadvantages of the main potential interventions (Section 3 and Table A1), tentatively rank them for various setting types (Section 4), and conclude by identifying important open research questions, investigation of which could improve these rankings and thus help further mitigate the threat of human‐engineered pandemics (Section 5).

What follows rests on expert opinion and relevant peer‐reviewed literature. We performed a thorough literature review on the described pathogen removal technologies, and we cite the most relevant and informative articles. Our paper aims to do more than merely start a discussion—it provides a structured framework for evaluating infectious aerosol management interventions, advances preliminary recommendations based on current evidence, and identifies critical research gaps that must be addressed to better prepare for aerosol‐mediated pandemics, especially those that could be man‐made with novel, engineered pathogens. To this end, we draw attention to the salient dimensions along which methods should be evaluated and then advance preliminary opinions on the best paths for mitigation. We also highlight how future research could shift these rankings, making the adoption of more effective paths more feasible and more ethical.

## 2 KEY CRITERIA FOR CHOOSING INFECTIOUS AEROSOL MANAGEMENT INTERVENTIONS TO MITIGATE ENGINEERED PANDEMICS

In the wake of COVID‐19, many groups have reviewed and recommended various infectious aerosol management strategies (Allen & Marr, 2020; Al‐Rikabi et al., 2023; ASHRAE, 2023; Bazant & Bush, 2021; Berry et al., 2022; Bueno de Mesquita et al., 2022; Chang et al., 2023; Elsaid & Ahmed, 2021; Ferrari et al., 2022; Izadyar & Miller, 2022; Nair et al., 2022; Xu et al., 2021; Yan et al., 2023). However, this guidance is limited to purely reactive strategies for preventing the spread of natural pathogens that are known to be circulating. Compared to reactive approaches for naturally occurring pathogens, proactive strategies for preventing the spread of engineered pathogens must meet more demanding standards along several key dimensions.

First, these proactive strategies must be more effective. Engineered pathogens could be expected to be no less harmful than natural pathogens. Indeed, they are likely to be much more harmful and more evasive to vaccines that have already been developed or can be developed quickly. They are likely to be far more infectious, and infectious pre‐symptomatically, than natural pathogens. They will cause infected people to shed more viable pathogens into the air, or they will require very little inhalation exposure to become infected. In light of these considerations, strategies must be capable of inactivating or removing engineered pathogens even more quickly and completely than strategies for inactivating and removing natural pathogens.

Second, proactive strategies must be less costly to run. Because reactive strategies are deployed only infrequently, for relatively short periods, and in locations that provide essential services during a pandemic, high daily operation costs can be tolerated. However, because proactive strategies must be deployed nearly every day and everywhere that people share indoor air, daily operation costs must be limited.

Third, exposure to interventions deployed preemptively must impose fewer health risks and other social costs. While short‐term exposure to some interventions used reactively may not lead to significant health risks, chronic exposure to the strategies used proactively may lead to serious health risks. Likewise, even if short‐term exposure to aesthetically unappealing or noisy strategies is tolerable for short periods, it may not be tolerable for frequent and long‐term applications.

Fourth, preemptive interventions can only be effective if they are implemented. Which interventions are feasible may depend on public perceptions of the threat of pathogens and other airborne contaminants, and their perceptions and acceptance of the technologies to ameliorate them. These perceptions may also change over time. People may initially appreciate the benefits and relative safety of novel interventions used temporarily during a pandemic, but they may lack such an appreciation of their benefits and safety when used indefinitely.

Fifth, preemptively deployed interventions must be more robust to human error, including failure to use or maintain the technology in ways necessary to reap their benefits. Human motivation to bear the costs of maintaining the installed technologies may also wane over time, especially when threats are no longer salient.

## 3 ADVANTAGES AND DISADVANTAGES OF THE MAIN INFECTIOUS AEROSOL MANAGEMENT INTERVENTIONS

We now describe the main indoor aerosol management interventions and their key advantages and disadvantages against deliberate pathogen pandemics. We divide the interventions into three categories: (1) ventilation, (2) air filtration, and (3) air disinfection. Each aims to reduce airborne infection risk by reducing the concentration of viable pathogen particles in people's breathing zones. For a summary of how each intervention fares along the most important dimensions, see Table A1.Throughout, our discussion will largely focus on how many equivalent air changes per hour (eACHs) can be achieved by each strategy. A strategy achieves N equivalent air changes (relative to a particular pathogen in a room of a particular size) when it reduces the concentration of viable pathogens by the same amount that ventilation would if it supplied the room with air that is free of viable pathogens at a rate of N x V (V = the volume of room) at well‐mixed conditions. For costs associated with achieving increasing eACHs, including high levels that are particularly important against highly transmissible pathogens like measles and engineered pathogens (Azimi et al., 2020; Mikszewski et al., 2021; Mikszewski et al., 2022; Nardell et al., 1991), see Table A2.

### 3.1 Ventilation with outdoor air

One way to reduce the concentration of viable pathogen particles in people's breathing zones is through ventilation introducing larger amounts of outdoor air. Natural ventilation relies on cross‐ventilation and/or stack (chimney) effect and can be accomplished by opening doors or windows in certain locations (Bramiana et al., 2023; Cho et al., 2012). The stack effect can be achieved by strategically opening windows on lower and upper levels to allow colder air to enter at lower levels and for warmer air to escape through upper levels (U.S. Department of Energy, n.d.). Windows can also be modified to increase ventilation (Fusaro et al., 2020; Lim & Kim, 2018; Zhang et al., 2022). In addition, gaps in window sills or cracks in building envelopes provide some continuous passive ventilation. Mechanical ventilation uses engineered systems to provide a flow of outside air into a building and it can be accomplished by using window fans, exhaust ventilation, or heating, ventilation, and air conditioning (HVAC) systems. Thus, the proportion of indoor air with an outdoor origin is influenced by both passive and active ventilation. It could be controlled by the number and duration of open windows and doors and also via HVAC system settings. The latter could be adjusted to bring in a certain ratio of (fresh) outdoor air to recirculating air (Chang et al., 2023).

The main *advantage* of ventilation with outdoor air is that it often has low upfront costs. Such costs tend to be low in shared spaces that already have either HVAC systems or operable windows and doors, which can be modified at low cost to increase airflow (e.g., by installing exhaust fans or adding screens so windows can fully open without compromising safety).

Further advantages of ventilation are that—under certain conditions—it can have both low ongoing costs and high effectiveness. Ongoing costs can be low in mild climates where outdoor air does not need to be pushed at high rates through air ducts or conditioned to maintain comfort. High effectiveness can be achieved in spaces with larger windows and doors, structural features that allow for inexpensive modifications (Escombe et al., 2019), and high airflows (Gładyszewska‐Fiedoruk & Gajewski, 2012) or fast air movement (e.g., cars driving at 25 miles per hour [Kim et al., 2023], or trains moving at 35 miles per hour [Shinohara et al., 2021]). For example, Escombe et al. (2007) found that opening windows and doors in eight hospitals resulted in a median of 28 air changes per hour, over twice the 12 air changes per hour standardly achieved by mechanical ventilation in high‐risk hospital environments, with higher ventilation rates in older buildings with larger windows and doors. Qian et al. (2010) demonstrated ventilation rates between 11.9 and 69.0 air changes per hour in other hospitals, while Escombe et al. (2019) demonstrated ventilation rates between 15 and 66 air changes per hour in hospital waiting rooms. Du et al. (2020) achieved 14–15 air changes per hour in university classrooms in the midst of a tuberculosis outbreak, resulting in a 97% reduction in the number of tuberculosis infections among close contacts. Van Dyke et al. (2022) found up to 22 air changes per hour on a school bus with windows and ceiling hatches open, and the defroster running.

Even in suboptimal conditions, natural ventilation can be effective to some degree. Thornton et al. (2022) conducted a systematic review of 20 studies of aerosol transmission of SARS‐CoV‐2. The review included 16 modeling studies, using inpatient and outpatient settings; three experimental studies, and only one observational study. Using these diverse settings, all 20 studies demonstrated positive results due to natural ventilation regardless of the outcome metric used: transmission rates, aerosol persistence, virus concentration, risk of infection, probability of infection, risk of cross‐infection, or longer exposure times before risk was deemed unacceptable. Thus, although largely based on modeled results, one may conclude, as did these authors, that some fresh air ventilation is almost always better than none, and pathogens of any size and type in a building's air space will be reduced, proportional to the degree of pathogen‐free outdoor air brought inside and mixed with potentially contaminated indoor air (For review, see Thornton et al. [2022]).

Even if this intervention typically cannot remove pathogens at the rates required to completely prevent the spread of a highly transmissible pathogen, any reduction in concentration of infectious quanta is desirable as that reduces exposures and the likelihood of exposure. In addition, it will reduce the buildup of CO_2_ and other pollutants (Lee et al., 2023), which may have cognitive benefits for occupants (Allen et al., 2016; Wargocki, 2000).

The “low tech” nature of opening a window or turning on a fan or the HVAC system makes them familiar and non‐threatening to occupants, and may provide a further comforting degree of individual control (Brager et al., 2004).

*The main disadvantages* of ventilation via outdoor air include that, in most places, ongoing costs are high, human cooperation is low, and environmental conditions are not consistently suitable for high rates of natural ventilation. First, the cost of constantly moving large volumes of outdoor air (in the case of mechanical ventilation) and constantly conditioning (i.e., heating, cooling, and humidification) large volumes of outdoor air (in the case of both natural and mechanical ventilation) will be an important limitation of this approach in many, if not most, climates with hot summers and cold winters (Faulkner et al., 2022).

Second, occupants with different temperature preferences or needs may disagree on the amount or duration of window or door openings, generating coordination challenges.

As it is, many people are dissatisfied by the temperature of the office buildings they occupy and believe it interferes with their ability to work (Graham et al., 2021). Individual inclinations to open windows fluctuate significantly as a function of temperature and relative humidity (Liu et al., 2022), and there is significant interpersonal variation in window‐opening tendencies even within the same climate (Niu et al., 2022).

Third, occupants may avoid opening windows out of concerns about noise pollution (Fusaro et al., 2020), air pollution (Niu et al., 2022), risk of falling out (Toprani et al., 2018), and the intrusion of insects and other creatures from outdoors (Faheem et al., 2022). Ventilation can introduce indoor air pollutants of outdoor origin in areas with significant ambient pollution, including pollutants from wildfires and other combustion sources, car emissions, and other stationary and mobile sources (Thurston et al., 2017).

A further limitation of ventilation is that many office buildings, especially recently constructed buildings, lack operable windows or mechanical ventilation systems that can deliver sufficient amounts of outdoor air.

### 3.2 Air filtration

Another approach to removing pathogens from the air is by filtering them out. Single viruses are generally in the range of 0.05–0.1 microns (µm) in diameter, although they are often attached to larger particles of dust or mucous materials. For reference, bacteria are generally 10 times larger, about 1 µm, and a human hair is 100 µm.

The efficiency of different filters is rated as minimum efficiency reporting values (MERV) and ranges from 1 to 20, with higher numbers corresponding to higher efficiencies. MERV rating is assigned based on filter performance in three different air particle size ranges, as defined by the American Society of Heating, Refrigeration, and Air Conditioning Engineers (ASHRAE, 2015). Filters that are capable of removing 99.97% of 0.3 µm particles are designated as high efficiency particulate air (HEPA) (ASHRAE, 2015;  U.S. Environmental Protection Agency, Office of Air and Radiation, 2019), with 0.3‐µm diameter designated the “most penetrating particle size” (MPPS). Particles that are larger or smaller than MPPS are captured by HEPA filters with an even higher efficiency than 99.97%, so smaller particles are not necessarily more resistant to filtration (ASHRAE, 2015). Filters with MERV rating of 17 largely fulfill requirements of HEPA filters.

During the COVID‐19 pandemic, many indoor space filters were replaced with higher rated MERV‐13 filters, which remove less than 75% of particles between 0.3 and 1 µm and >90% of particles greater than 1 µm. Since many virus particles are adsorbed to larger particles, even a MERV‐13 filter can have a substantial beneficial effect. MERV‐13 is the rating often recommended for hospital operating rooms, inpatient facilities, superior commercial buildings, and other areas that need to be clean but not absolutely sterile. To filter out infectious airborne particles, ASHRAE recommends that organizations use MERV‐13 or the highest achievable filtration level (The American Society of Heating, Refrigerating and Air‐Conditioning Engineers, n.d.). MERV‐14 or higher rated filters remove >75% of 0.3 um particles. However, more efficient filters are not always preferable, for reasons explained below.

The filter ratings refer to the properties of the filtration material. The actual removal of airborne particles, including infectious particles, depends on the volume of air filtered per unit of time in relation to the indoor space volume (e.g., number of air changes per hour) and the introduction of new particles into the indoor space. For example, in a lab experiment, a single air change with a HEPA filter cleared only 85% of SARS‐CoV‐2 particles, but after 7 air changes (35.5 mins), 99.97% were cleared (Ueki et al., 2022).

We shall discuss three main categories of air filtration: (a) HVAC and in‐duct (which range from low MERV to high MERV and HEPA); (b) PACs; and (c) DIY air cleaners.

#### 3.2.1 High‐efficiency HVAC filtration

HVAC systems include air handlers, ducts, fans, filters, heating, and cooling coils. In addition to increasing the amount of outdoor air introduced by the HVAC system (see previous section), the concentration of viable pathogen particles can be reduced by improving the filtration within the air handler or ducts from, for example, MERV‐8 to MERV‐13 or better.

One main *advantage* of improving filtration within an HVAC system's air handler or ducts is that it can be easily implemented in many buildings with existing HVAC systems, and that it can often be at least as effective and less costly than ventilation with outdoor air. In a systematic review by the same group as above, 23 studies addressing the question of in‐duct HVAC filtration efficacy for virus removal showed that use of filters was associated with increased virus removal from air and decreased transmission and infection risk, but that there was limited benefit to filters rated above MERV‐13 (Thornton et al., 2023). Although costs increase with higher efficiency filtration (Faulkner et al., 2022), they remain lower than the cost of using ventilation alone to achieve comparable reduction in transmission risk (Azimi & Stephens, 2013; Chang et al., 2023; Thornton et al., 2023; Zaatari et al., 2023). Generally, mechanical ventilation using MERV‐13 is more cost‐effective than using MERV‐10, HEPA, or ventilation with outdoor air—except for mild climates like San Diego, in which significant energy does not need to be used to heat or cool air brought in from outdoors (Chang et al., 2023; Faulkner et al., 2022; Zaatari et al., 2023).

Another significant advantage of improving filtration within the HVAC system is that doing so will not only remove a substantial fraction of infectious particles (providing increased protection during pandemics or seasonal influenza events), but also provide cleaner air during outdoor pollution events, such as during wildfires, while significantly reducing the concentrations of indoor allergens (Shin et al., 2023; Thornton et al., 2022).

A final advantage is that improving filtration carries a low risk of human error. While filters must be installed correctly, filter replacement is straightforward once any necessary adjustments to the HVAC required to accommodate the more efficient filter have been made. And because the filters are mounted within the HVAC system, they are typically inaccessible and invisible to building occupants, making them more easily accepted, aesthetically appealing, and harder to interfere with or damage than other exposed devices.

High‐efficiency HVAC filtration has *disadvantages* as well. Most importantly, there are limits to how much it can reduce transmission risk. First, there are limits to where it can be implemented. The air movers (e.g., blowers) in most existing whole‐building air handling systems are insufficiently powerful to accommodate the increased resistance of a HEPA filter, and often even of MERV‐13, without resulting in excessive and costly pressure drop (Faulkner et al., 2022). Installing high‐efficiency filtration beyond that which a system is designed, might render the system inoperable or result in such slow movement of air that the rate at which pathogens are removed does not increase or perhaps even decreases (Faulkner et al., 2022; Zaatari et al., 2014), compared to installing lower efficiency filtration. Changes in airflow rates due to installation of a more efficient filter change may also affect occupant comfort (Liu et al., 2017).

Second, even in spaces where high‐efficiency filtration can be implemented effectively, there are limits to how much it can reduce transmission risk. In‐duct filters remove pathogens from the air only after they have traveled across the room into the air ducts, potentially infecting occupants along the way (Cadnum et al., 2022; Pantelic & Tham, 2013). But more importantly, it is only with powerful, expensive HVAC systems found in hospitals, typical operating rooms, and ultra‐clean operating rooms that high‐efficiency filters can be used to achieve 12, 25, or 69 (equivalent) air changes per hour, respectively (Gormley et al., 2017; Lans et al., 2024, 2023; Pereira et al., 2020). Improving filtration in less powerful systems can only do so much to reduce transmission risk. For instance, in one simulation study of influenza transmission in a hypothetical office space, the benefits of MERV‐15 filtration were equivalent to only 1.37 additional outdoor air changes per hour, reducing relative risk of infection by less than half (Azimi & Stephens, 2013). In another simulation study, upgrading to MERV‐13 or HEPA reduced measles transmission risk in schools by only 28% and 33%, respectively (Azimi et al., 2020).

Consequently, while many existing HVAC systems can accommodate MERV‐13 filters and upgrading from MERV‐7 to MERV‐13 may reduce transmission risk to some degree at low cost (Azimi & Stephens, 2013; Das et al., 2023), combatting highly transmissible pathogens would require improving filtration beyond MERV‐13 without a counterproductive pressure drop (Faulkner et al., 2022). However, the upgrades needed to improve filtration further are among the most expensive interventions we discuss, both in terms of upfront costs and energy consumption (Abboushi et al., 2022; Azimi & Stephens, 2013; Cai et al., 2022; Faulkner et al., 2022, 2024). While integrating powerful HVAC systems into new construction can be more feasible, their costs can remain prohibitively expensive (Cacciari et al., 2004; Gormley et al., 2017; Lans et al., 2023).

#### 3.2.2 Portable air cleaners

PACs are self‐contained commercially available devices that consist of one or more fans and one or more filters, one of which is a HEPA filter. Some PACs have additional filters that include coarse pre‐filters to remove larger particles and debris or activated carbon filters to remove volatile organic compounds (VOCs).

PACs come in different capacities to clean rooms of different sizes. Manufacturers typically provide their clean air delivery rates (CADRs) or a room size for which they are suitable. Typical users are individual homeowners/renters and occupants of office spaces who wish to supplement the air‐cleaning capacity of their central HVACs. For larger spaces, multiple smaller PAC units instead of one or two large units are preferable both in order to minimize the distance pathogens must travel before being removed by the cleaner and to avoid creating airflows that expose occupants near the cleaners to a larger number of pathogens (Bluyssen et al., 2021; Coyle et al., 2021; Derk et al., 2023).

The main *advantage* of PACs is high cost‐effectiveness. With a CADR of 300 cubic feet per minute (CFM), a typical PAC can provide 10 eACHs in a 15 × 15 × 8 foot room, while two PACs can provide 10 eACHs  in a room twice that size (Allen & Ibrahim, 2021; Myers et al., 2022). Simulation studies suggest that operating just one 400 CFM PAC (or one 328 CFM PAC) for every 500 square feet of floor space reduces risk of measles infection (or COVID‐19 infection) just as much, if not more, than HVAC systems with filtration (Azimi et al., 2020; Shen et al., 2021). Studies in real‐world settings confirm that PACs can efficiently reduce infectious aerosols (Curtius et al., 2021; Das et al., 2023; Duill et al., 2021; Myers et al., 2022; Vartiainen et al., 2024) and surrogates of infectious aerosols (Derk et al., 2023; Ren et al., 2021), and that air changes achieved via PACs are just as effective, if not more effective, at reducing exposures to aerosols (Coyle et al., 2021).

Because the efficacy of each properly positioned PAC is independent and additive (Noh & Yook, 2016), using more PACs, or more powerful PACs, to achieve a higher CADR per square foot may reduce infection risk significantly more than upgrading HVAC filtration to MERV‐13 (Azimi et al., 2020). For example, J. H.Lee et al. (2022) used multiple PACs to add 25 eACHs to a single‐bed hospital room.

At the same time, because there is no need to push air through ductwork (Faulkner et al., 2024), PACs require less energy to filter a volume of air than HVAC systems with high‐efficiency filters (Noh & Yook, 2016).

A further advantage of PACs is that, like efficient HVAC filtration, they efficiently reduce concentrations of non‐infectious indoor air particulates (Han et al., 2022; Lu, Laumbach, et al., 2024; May et al., 2021; Xiang et al., 2021), which may reduce the risk of acute and chronic cardiopulmonary disease (U.S. Centers for Disease Control & Prevention, 2024b; Fisk & Chan, 2017a, 2017b) and mental illness (Taylor et al., 2021).

The main *disadvantages* of PACs are their high initial cost (Pistochini, 2021; Product Finder—ENERGY STAR Certified Room Air Cleaners, n.d.), maintenance costs, noise (especially at higher fan settings) (Bluyssen et al., 2021), and susceptibility to human error (e.g., using lower settings to avoid noise [Granzin et al., 2023]; moving them to less efficacious locations in the room [Novoselac & Siegel, 2009]). Because there is no convenient or affordable way to measure the filter loading to ascertain that a replacement is needed, filter replacement is typically schedule‐based instead of need‐based (e.g., following load or pressure drop) increasing user cost and potentially reducing effectiveness if the schedule does not account for heavy filter loading. Lack of knowledge among non‐experts and deceptive marketing can lead some to underestimate the efficacy of HEPA filters and overestimate the efficacy of alternative products, leading to reduced adoption of HEPA PACs (Tufekci, 2023). These factors can also lead people to purchase air cleaners that are less effective than advertised (Noh & Oh, 2015).

PACs may be less effective depending on placement, and they could concentrate aerosols in certain locations depending on airflow (Castellini et al., 2022). Indeed, in cases where direct transmission cannot be prevented (e.g., kindergartens), they may not prevent spread at all (Falkenberg et al., 2023).

#### 3.2.3 Do‐it‐yourself air cleaners

DIY air cleaners typically consist of a box fan and one or more filters connected into a single unit, often by using duct tape. Different designs include a single filter + box fan, two filters in a triangle formation, and a Corsi‐Rosenthal box (four or five filters + box fan) (Holder et al., 2022; Myers et al., 2023; Srikrishna, 2022).

The main *advantage* of DIY air cleaners is that they provide high CADR values at very low initial cost. DIY air cleaners cost <$50 for single‐filter varieties and <$100 for multi‐filter varieties. Performance in terms of CADR depends on the filter MERV rating, filter thickness, and filter manufacturer (Derk et al., 2023; Myers et al., 2023). Lab tests show that for PM_2.5_ mass concentration, single‐filter DIY air cleaner configurations can achieve CADR of 150–250 CFM. Two‐filter configurations achieve CADR of close to 400 CFM and four filter configurations achieve CADR of ∼450 CFM. Other studies reported CADR as high as 600–850 CFM for a five‐panel Corsi‐Rosenthal box (Dal Porto et al., 2022). These CADR values are comparable to or exceed commercial HEPA portable cleaners (Eykelbosh, 2023).

In addition to low upfront costs, ongoing costs are affordable. Energy consumption of a popular box fan is ∼80 W (Myers et al., 2023). Assuming the cost of electricity is $0.15/kWh and the cleaner is operated for 12 h/day for 30 days, the cost would be ∼$4.50, approximately a cup of coffee (U.S. Energy Information Administration, n.d.). Thus, the total annual cost would be about $50 for electricity and ∼$30–$40 for new filters, if replaced every 6 months.

Like other forms of filtration, DIY air filtration has the ability to decrease other forms of indoor air pollution (Dodson et al., 2023; Manz et al., 2023).

Deploying DIY air cleaners has several *disadvantages* on top of inheriting the disadvantages of PACs. First, it is effective only to the extent that lay members of the public are willing to build DIY cleaners with proper care. Although instructions for building DIY cleaners are straightforward and easy to follow, it cannot be guaranteed that everyone in a position to build a DIY cleaner will do so and do so diligently. The noise of DIY air cleaners can be higher than even that of commercial PACs, especially for single‐ and double‐filter configurations, which may cause users to lower fan speed—and CADR—or turn it off altogether. However, air cleaners made with personal computer (PC) fans—some of which can be purchased pre‐assembled—can move just as much air more quietly than both traditional DIY air cleaners and most PACs (Intertek, 2023a, 2023b; Rosenthal, 2023). Although many builders choose to “beautify” DIY air cleaners by adding color or ornaments, DIY air cleaners are usually bulkier and less aesthetically pleasing than commercial PACs, which could further reduce adoption. Space requirements could also be an issue in tight apartments, offices, or classrooms. There are no indicators that a filter needs to be replaced; thus, a schedule‐based replacement must be followed.

### 3.3 Air disinfection

In addition to ventilation and filtration, strategies to kill or inactivate airborne pathogens are viable, and potentially more efficient and cost‐effective. Different techniques for disinfection use different chemical disinfectants or radiation, which can be applied simultaneously to different locations in a building.

Below we discuss three specific applications of ultraviolet irradiation: upper‐room UVC, direct far‐UVC, and in‐duct UVC. We also consider a chemical disinfectant, TEG. There are alternatives beyond UVC and TEG. However, the National Air Filtration Association (NAFA) has stated that ionizers, ozone generators, plasma, and other air cleaning technologies have not been proven to reduce infection in real buildings, even if they may show promise based on tests performed in idealized laboratory environments (National Air Filtration Association, 2021). Many of these, along with other technologies we review that add or generate hydrogen peroxide, hydroxyl radical, reactive oxygen species, and PM_2.5_, are considered too toxic for widespread use (National Academies of Sciences, Engineering, and Medicine, Division on Earth and Life Studies, Board on Chemical Sciences and Technology, & Committee on Emerging Science on Indoor Chemistry, 2022).

#### 3.3.1 Upper‐room UVC

In typical applications of upper‐room UVC, low‐pressure mercury lamps emit 254 nm UV light across unoccupied portions of indoor space, either above 9 feet or between 8 and 9 feet with built‐in louvers used to prevent the light from entering occupied portions of the space (U.S. Department of Health & Human Services Centers for Disease Control & Prevention; National Institute for Occupational Safety & Health [NIOSH], 2009). When airborne pathogens in those upper‐room areas receive a sufficient dose of 254 nm light, their DNA is damaged, and they are inactivated (Spicer, 2021). With sufficient air mixing induced by body heat, a ceiling fan, or an HVAC system, large volumes of air from upper regions of the space, where the pathogens have been inactivated, are delivered to lower regions of the space, substantially decreasing the presence of viable airborne pathogens throughout the space.

Upper‐room UVC was first used to disinfect air close to 100 years ago, with initial deployments in hospital operating rooms, infant wards, and schools (Reed, 2010). Despite early studies suggesting high efficacy in the prevention of airborne illnesses like measles (Wells, 1943), upper‐room UVC has been mostly neglected following the development of antibiotics, immunizations for common viral infections (Brickner et al., 2003), along with failures to replicate early successes. These failures have since been attributed to a failure to prevent occupants from infecting one another in other contexts (e.g., school buses) where UVC was not being used to disinfect shared air (Nardell & Nathavitharana, 2019; Reed, 2010). In the last few decades, there has been renewed interest in using UVC to control the spread of tuberculosis. This has led to a new wave of research on the safety and efficacy of using upper‐room UVC to disinfect indoor air.

The main *advantage* of upper‐room UVC is its high efficacy. The rate at which upper‐room UVC delivers viable‐pathogen‐free air to occupied spaces will depend on many factors, including the number and placement of UVC fixtures (e.g., mercury lamps), relative humidity, air mixing, and the type of pathogen being inactivated. But upper‐room UVC typically delivers viable‐pathogen‐free air at rates far higher than the intense ventilation found in hospital patient rooms. In hospital‐room‐sized chambers, laboratory studies have shown that upper‐room UVC can reduce viable vaccinia viruses, poxviruses, and SARS‐CoV‐2 at rates of 10, 20, 50, or more eACHs (First et al., 2007; Innovative Bioanalysis, 2021; Ko et al., 2002; Landry et al., 2025; Linnes et al., 2014; McDevitt et al., 2008; Riley et al., 1976; Rudnick et al., 2015; Walker & Ko, 2007). Studies have shown that upper‐room UVC in real hospital rooms can reduce infection from *Mycobacterium tuberculosis* (Escombe et al., 2009), inactivating the pathogen at a rate of 24 eACHs (Mphaphlele et al., 2015), which is similar to air change rates in operating rooms. It has also been shown to decrease the risk of infection from other airborne diseases in a hospital setting (Ethington et al., 2018).

Importantly, the susceptibility of *Mycobacterium tuberculosis* to inactivation by external means, such as UVC light, is lower than the susceptibility of enveloped viruses like SARS‐CoV‐2 and influenza; that suggests that UVC‐254 light can be effective against engineered viruses with pandemic potential in real‐world settings (Beggs & Avital, 2020; Beggs et al., 2006; McDevitt et al., 2012). Real‐world studies confirm that upper‐room UVC in schools has been effective at preventing the spread of such viruses, significantly reducing cases of measles, mumps, and chickenpox (Bahlke et al., 1949; Perkins et al., 1947; Reed, 2010; Wells et al., 1942). A recent analysis of historical data reveals that upper‐room UVC can reduce student absences due to respiratory illness in general by between a third and a half (the only exception is summer, perhaps because students spend more time outdoors and spread is lower anyhow) (Ryan, 2023).

A second advantage of upper‐room UVC is that these high rates of disinfection are achieved at relatively low cost, making it highly cost‐effective. A single louvered UVC fixture costs between $900 and $1600 (Davidson, 2021), and delivers more eACHs than similarly priced PACs and HVAC. Some DIY upper‐room UVC setups can cost less than $200 per unit, making upper‐room UVC even more affordable (Davidson, 2021). Replacement bulbs and maintenance are comparable in price to replacement air filters for PACs and MERV‐13 filters and HVAC maintenance. Upper‐room UVC fixtures consume less energy than comparable in‐duct filtration and HVAC systems to achieve high levels of disinfection (Abboushi et al., 2022; Faulkner et al., 2024). In one study, achieving 1 eACH with upper‐room UVC was nearly 10 times less expensive than achieving it with ventilation or with any of three PACs (Nardell, 2021). The End TB Transmission Initiative estimates that when buying in bulk (10 units), the initial costs and recurring costs of each unit together come to approximately $300 per year (End Tuberculosis Transmission Initiative & Stop TB Partnership, 2019).

A third advantage of upper‐room UVC is that there is a very low risk of dangerous, long‐term exposure to UVC light. Because ultraviolet light is invisible, occupants can in principle be exposed to hazardous levels of UVC light without realizing it (Kowalski, 2009). However, even with high levels of irradiation in unoccupied regions of the room, occupants are exposed to exceedingly low levels of UVC light when reflection into occupied regions is properly minimized to levels far below ACGIH threshold limit values (TLVs) set by the American Conference of Governmental Industrial Hygienists (ACGIH) (First et al., 2005). Moreover, because almost none of this UVC light makes it to the basal layer of the epidermis, “real risk of UV photocarcinogenesis at 254 nm is extremely small,” (Sliney, 2013) far lower than the risk of photocarcinogenesis from the ultraviolet‐B (280–315 nm) radiation present in sunlight (Forbes et al., 2021).

According to Sliney and Stuck (2021), the risk of daily exposure below ACGIH TLVs “is comparable to only a few minutes outdoors in sunlight during late spring or early summer” (p. 489), a risk people rationally tolerate in order to secure benefits far smaller than the avoidance of infection. The International Commission on Illumination estimated that, in a “worst‐case scenario” in which someone was exposed to 254 nm UVC light at the International Commission on Non‐Ionizing Radiation Protection (ICNIRP) 8‐h exposure limit (or the pre‐2022 ACGIH TLV) of 6 mJ/cm^2^ per day—more than three times the highest maximum occupant exposure in M. W. First et al. (2005)—over 8 h a day, 5 days a week, for 20 years, their risk of non‐melanoma skin cancer would increase only by a factor of .37% (International Commission on Illumination 2010, p. 6). On the basis of this and other data, Forbes et al. (2021) conclude that “The skin cancer risk associated with responsible use of germicidal UVR [ultraviolet radiation] is too small to be detected or anticipated from the data and is certainly vanishingly small relative to summer sunlight exposure or other imminent risks such as respiratory pathogen exposure.” (p. 482)

It follows that any long‐term risks of upper‐room UVC to skin or eyes would be experienced only if irradiation into occupied regions were not properly minimized with some combination of louvers (First et al., 2005; Milonova et al., 2016), lips (Davidson, 2021), anti‐reflective paints or ceiling tiles (Wengraitis & Reed, 2012), suspended egg‐crate ceiling tiles (Linnes et al., 2014), and measurement (Hou et al., 2021; Milonova et al., 2016). However, short‐term exposure at even low doses quickly generates effects—erythema and photokeratitis—that alert occupants to the need for adjustments long before long‐term exposure at high levels is possible (Sliney & Stuck, 2021). Nearly all known reports of accidental exposure have resulted in such transient but perspicuous effects, both in cases where people were accidentally exposed directly to high doses—because UVC lights used for surface disinfection were installed or used improperly (Boudet et al., 2023; Das et al., 2021; Emre et al., 2022; Gupta et al., 2021; Leung & Ko, 2021; Sengillo et al., 2021; Trevisan et al., 2006; Wang et al., 2021; Zaffina et al., 2012)—and in cases where the upper‐room UVC device was installed correctly and exposure was indirect (because the ceiling was reflective) (Moss & Seitz, 1991) or in which occupants entered the upper regions of the room (Brickner & Vincent, 2013; Nardell et al., 2008). Clinical effects were observed up to 2 years only in one case of extreme overexposure, in which nurses were exposed directly to 254 nm at close range, receiving a dose over 100 times higher than the ICNIRP limits at close range for an hour while preparing drugs under a laminar flow hood (Zaffina et al., 2012).

A final advantage of upper‐room UVC is the complete absence of noise, aside from any mechanical means of ensuring sufficient air mixing.

Upper‐room UVC has some limitations and *disadvantages*. One main limitation is that, in order to avoid exposing occupants to high doses of UVC, it can only be used safely in rooms with high ceilings (above 8 feet; ideally above 9 feet) (NIOSH 2024a, 2009). In spaces with lower ceilings, occupants would have to wear specialized suits and goggles to protect their skin and eyes. Also, upper‐room UVC has proven less efficacious at high relative humidity under laboratory conditions, although it has so far remained efficacious in real‐world settings that are humid (Nardell et al., 2013).

Upper‐room UVC also has some other disadvantages. First, when lamp fixtures are installed improperly or safety precautions are ignored, there is a risk of short‐term damage to exposed skin or the sclera and conjunctiva of the eye, leading to irritation and skin burns (Blatchley et al., 2023; Glaab et al., 2021; Welch et al., 2018). However, as argued above, the tendency to produce acute but short‐lived side effects can also be seen as an advantage in that it will immediately prompt adjustments to prevent chronic, high‐dose exposure that could lead to delayed effects like skin cancer.

Another disadvantage that has been appreciated only more recently is that upper‐room UVC can produce harmful indoor air pollution, especially in settings with high ventilation rates and high levels of ozone in outdoor air, via indoor ozone chemistry with VOCs and UV photolysis of VOCs (Graeffe et al., 2023; Peng, Miller, et al., 2023). To minimize these disadvantages, upper‐room UVC must be installed by trained professionals and supplemented by air cleaners with carbon filters that mitigate ozone and VOCs produced by the upper‐room UVC.

Some have suggested that if using upper‐air UVC necessitates some form of particulate and/or air filtration to avoid indoor air pollution, then air filtration alone should be used to manage infectious aerosols (Graeffe et al., 2023; Jimenez, 2023). However, the amount of air filtration required to mitigate indoor air pollution created by the upper‐room UVC is far lower than the amount of ventilation needed to prevent the spread of highly transmissible engineered pathogens with pandemic potential if ventilation alone is used. According to one study modeling how measles would spread across schools if students were unvaccinated, even an aggressive approach using traditional methods—upgrading to HEPA filters, significantly increasing ventilation rates, and using 800 CFM PACs *simultaneously*—would leave students at a risk 30 times higher than if they were instead vaccinated and provided ordinary levels of ventilation and filtration (Azimi et al., 2020). Clearly, in the event of an engineered pathogen that spreads like measles and against which the population is unvaccinated, we will need more effective approaches like upper‐air UVC.

#### 3.3.2 Direct far‐UVC lighting

Like 254 nm UVC, direct far‐UVC (222 nm) light inactivates a wide variety of pathogens (Buonanno et al., 2020; Eadie et al., 2022; Ma, Bright, et al., 2023; Narita et al., 2020; Song et al., 2023; Taylor et al., 2020; Wang et al., 2023). However, far‐UVC has limited ability to penetrate living cells in the skin and eyes and to generate the kind of damage that typically precedes skin cancer (Barnard et al., 2020; Blatchley et al., 2023; Busch et al., 2023; Conneely et al., 2022; Finlayson et al., 2022; Hickerson et al., 2021; Narita et al., 2018; Zamudio Díaz et al., 2023), allowing its use even in occupied spaces without causing any acute side effects on skin and eyes (Buonanno et al., 2017; Eadie, Barnard, et al., 2021; Eadie, O'Mahoney, et al., 2021; Fukui et al., 2020; Kaidzu et al., 2021; Kousha et al., 2024; Narita et al., 2018; Sugihara et al., 2023). In typical applications of direct far‐UVC, filtered krypton‐chloride excimer lamps emit 222 nm UV light throughout both unoccupied and occupied portions of indoor space. For these reasons, much contemporary scholarship is excited about direct UVC light as a strategy for infectious aerosol management (Bergman et al., 2021; Blatchley et al., 2023; Brenner, 2023; Bueno de Mesquita et al., 2022; Esvelt, 2022).

Since far‐UVC inactivates pathogens, but can be used in occupied portions of indoor space without generating acute side effects, it has two significant *advantages* beyond the high efficacy of all UV light solutions. First, unlike upper‐room UVC, direct far‐UVC can be used to disinfect spaces with low ceilings and poor air mixing. Second, because it can be used to irradiate air across an entire room, it can also inactivate a variety of airborne pathogens even more quickly than upper‐air UVC and at the height at which occupants inhale air (Buonanno et al., 2017, 2020, 2024; Eadie et al., 2022; Wood et al., 2022). For example, Eadie et al. (2022) used 222 nm in an unoccupied, well‐ventilated room‐sized chamber to inactivate aerosolized *Staphylococcus aureus* at an average rate of 35 equivalent air changes per hour, which remains significantly higher than the other strategies (with the exception of upper‐room UVC and, as we will see, TEG). They achieved these high rates of disinfection by using 222 nm UVC with intensity low enough that if any occupants were exposed to the UVC for 8 h, their skin or eyes would receive a dose below the conservative ICNIRP 8‐h exposure limit of 23 mJ/cm^2^, as well as below the ACGIH's TLV before ACGIH made revisions in 2022 (American Conference of Governmental Industrial Hygienists, 2021, 2022; International Commission on Non‐Ionizing Radiation Protection (ICNIRP), 2004). When they used higher intensity 222 nm UVC that occupants could be exposed to for 8 h without exceeding the ACGIH's current TLV of 480 mJ/cm^2^ for skin, Eadie et al. inactivated *Staphylococcus aureus* at an average rate of 184 equivalent air changes per hour. Likewise, Buonanno et al. (2024) inactivated aerosolized murine norovirus in an occupied room at an estimated rate of 2960 equivalent air changes per hour (assuming poor air mixing), using 222 nm UVC at an intensity that occupants could be exposed to over 8 h while receiving a dose (79.5 mJ/cm^2^) far below ACGIH's current TLVs for skin (480 mJ/cm^2^) and eyes (160 mJ/cm^2^).

Despite direct far‐UVC's significant advantages, it also has significant *disadvantages*. First, it is expensive. The filtered KrCl excimer lamps required to disinfect 1000 square feet of space with 222 nm UVC cost between $10,000 and $20,000 per year (Esvelt, 2022), roughly 10–20 times the cost of a set of the most affordable mercury‐vapor lamps that emit 254 nm UVC (Davidson, 2021), or roughly two to three times the cost of a set of standard louvered fixtures that emit 254 nm (NIOSH 2024a; Kleinwaks et al., 2023). While KrCl excimer lamps can generally inactivate viruses more efficiently with a unit of power than mercury‐vapor lamps—both because that power is used to irradiate more air and because a unit of 254 nm inactivates coronaviruses (and likely other viruses) at twice the rate as an equal unit of 254 nm (Blatchley et al., 2023; Lu, Laumbach, et al., 2024)—using direct far‐UVC remains far more expensive than using upper‐air UVC. This is because a KrCl excimer lamp costs between $1 and $5 per mW of its output power, while a mercury lamp costs between $0.001 and $0.1 per mW of its output power (Blatchley et al., 2023). Moreover, KrCl excimer bulbs tend to need replacement more often than mercury‐vapor bulbs, and their efficiency at converting electricity into ultraviolet light is significantly lower than mercury‐vapor bulbs (Blatchley et al., 2023), such that they consume nearly as much electricity to disinfect viruses and more electricity to disinfect bacteria (Lu, Dong, et al., 2024).

Second, we cannot at this point be fully confident that long‐term, high‐dose exposure to direct far‐UVC has no negative health effects—especially at the high doses at which it would be most likely to halt the spread of engineered pathogens. While numerous studies reveal that exposure to 222 nm does not result in the kind of damage that typically precedes skin cancer, there have been few studies on long‐term far‐UVC exposure. In the only long‐term study on humans, Sugihara et al. (2023) found no adverse health effects on the eye or eyelid skin of six ophthalmologists who were exposed to far‐UVC over the course of a year. However, they were exposed to far‐UVC for an average of only 6.7 h a week, and they were exposed to doses of far‐UVC below the conservative ICNIRP 8‐h exposure limit (between 20 and 25 mJ/cm^2^) and pre‐2022 ACGIH TLV (23 mJ/cm^2^) (American Conference of Governmental Industrial Hygienists, 2021; International Commission on Non‐Ionizing Radiation Protection (ICNIRP), 2004). More human studies are necessary to elucidate any negative health effects (Görlitz et al., 2023; Hessling et al., 2021; Nishigori et al., 2023).

There are two long‐term studies on animals. In one, SKH‐1 hairless albino mice, who are susceptible to UV‐induced skin cancer, did not develop cyclobutane pyrimidine dimers (the DNA precursor lesions most commonly associated with UV‐induced skin cancer [Pfeifer & Besaratinia, 2012]) or other obvious abnormalities in skin after 66 weeks of exposure to 400 mJ/cm^2^ over 8 h each day, 5 days each week (Welch et al., 2023). Another study investigated the effect of far‐UVC on wild‐type hairless albino mice and photosensitive *Xpa*‐knockout mice, who are 10,000 times more susceptible to UV‐induced skin cancer due to their impaired ability to remove cyclobutane pyrimidine dimers. Wild‐type mice received 500 mJ/cm^2^, while *Xpa*‐knockout mice received 100 mJ/cm^2^ 3 days a week over 15 weeks of exposure. None of the mice developed any skin cancer or cyclobutane pyrimidine dimers, nor did they experience any damage to their lenses or retinas (Yamano et al., 2020). However, studies of other potential indicators of damage remain limited (Görlitz et al., 2023; Nishigori et al., 2023).

Furthermore, existing studies of other indicators leave open the theoretical possibility of long‐term effects. For example, one study confirms that delivering a total of 222 nm UVC at an intensity of 73 µW/cm^2^ over 60 min (equivalent to 262.8 mJ/cm^2^, per this paper's authors) does not generate pyrimidine dimers in human ARPE19 retinal cells, but it also found that it led to an increase of γH2AX nuclear foci, another indicator of DNA damage (Ong et al., 2022). It also found that cells exposed to 222 nm had decreased viability and growth. Another study suggests that, although exposure to 222 nm UV light at only 25 mJ/cm^2^ (approximating the pre‐2022 ACGIH TLVs) for 8 h a day, 5 days a week, for 8 weeks results in significantly less skin damage in hairless mice than direct exposure to 254 nm UV light, it produces some minor skin damage affecting skin generation (Tavares et al., 2023). While this skin damage is only detectable by lab techniques, it raises the possibility that chronic exposure to far‐UVC could result in premature skin aging (e.g., wrinkling). More studies on these and other potential indicators of damage at different doses of 222 nm are necessary to understand the long‐term effects of chronic far‐UVC exposure (Görlitz et al., 2023).

Even if chronic exposure to far‐UVC itself turns out to be safe for skin and eyes, far‐UVC may affect the airways because it creates gaseous and particulate air pollution indoors. This constitutes a third disadvantage. First, in regions where SO_2_ is produced by coal‐based heating and cooking, 222 nm results in the formation of sulfate nanoparticles (Liang et al., 2024). Second, it generates ozone (Barber et al., 2023; Link et al., 2023; Peng, Day, et al., 2023), especially when used in spaces cleaned with limonene‐based cleaners for heating and cooking (Brenner, 2024; Jenks et al., 2024). In addition to being harmful in itself, ozone may lead to the formation of harmful particulate matter—especially at the high fluence rates that deliver high rates of disinfection (Brenner, 2024; Jimenez, 2023; Ma, Burke‐Bevis, et al., 2023; National Academies of Sciences, Engineering, and Medicine, Division on Earth and Life Studies, Board on Chemical Sciences and Technology, & Committee on Emerging Science on Indoor Chemistry, 2022; Peng, Miller, et al., 2023). To avoid these harms, far‐UVC must be used with high ventilation or filtration to remove particulate matter or with activated carbon filters placed in HVAC ducts, PACs, or DIY air cleaners to remove secondary pollutants.

A fourth disadvantage of far‐UVC is that in societies where trust in institutions is low, users might not trust that far‐UVC is safe—perhaps misled by the safety issues with other UV wavelengths (Scientific Committee on Health, Environmental and Emerging Risks [SCHEER], 2017). This may lead them to avoid spaces where these systems are used or even to deliberately damage them. If so, less efficacious methods may turn out to be more effective in practice.

A fifth disadvantage of direct far‐UVC is that it degrades various types of building interiors, transportation interiors, and items—especially certain polymers and fabrics—changing their color and altering their mechanical properties in ways that make them less functional or more likely to break (Dalton et al., 2023; Drungilas et al., 2023; Mitxelena‐Iribarren et al., 2022; Ravi et al., 2023; Teska et al., 2020;  U.S. Plastic Corp., 2023). American Ultraviolet, a manufacturer of UVC fixtures, estimates that long‐term UVC exposure reduces the lifespan of plastic items by 10% (American Ultraviolet, n.d). Direct far‐UVC also harms at least some plants (Otake et al., 2021).

#### 3.3.3 In‐duct UVC

Broad spectrum UV light, which has been known for decades to be effective for disinfection of surfaces within the HVAC system, may also disinfect the air passing through the system if airflow is slow enough to afford UV light enough time to deactivate or kill the pathogens in that air. When the intensity of the UV bulbs and length of the irradiated area are known, the dwell time of the air passing through that area of the HVAC system can be calculated for energy needed to damage viruses delivered on each pass. With reports of how susceptible each pathogen is to UV light, the efficacy of the in‐duct system can be calculated. However, even then, in‐duct UV lights require safeguards to ensure the UV light is confined only to the space within the ductwork, as light leakage can create the skin and eye problems mentioned above.

A key *advantage* of in‐duct UVC is that it can achieve high disinfection efficiency in HVAC systems that cannot accommodate high‐efficiency filters like MERV‐13 (see 2a above) (Luo & Zhong, 2021; Yang et al., 2018). It also uses comparable or less energy than achieving the same efficacy by upgrading to MERV‐13 (Faulkner et al., 2024; Ye et al., 2024a, 2024b, 2025). When installed in a central air handler supplying recirculated air to an entire floor or building, it can deliver a unit of pathogen‐free air at a lower cost per eACH than PACs or DIY air cleaners (see Table A2).

Additionally, because it is not in the room, the risk of harmful short‐term exposure is lower than that of upper‐room UVC, and the risk of harmful long‐term exposure is lower than that of direct far‐UVC. In‐duct UVC is also less vulnerable to accidental or deliberate damage than placing technology near occupants.

A key *disadvantage* of in‐duct UVC is that, like in‐duct filtration, its ability to quickly disinfect large amounts of air is limited by the ability of the HVAC system to ensure sufficient airflow (Nardell et al., 2013). Moreover, air must in any case move relatively slowly through ductwork to ensure pathogens receive a sufficient dose of UVC (Zhang & Lai, 2022). These limitations can result in relatively few eACHs compared to in‐room technologies, even if the air treated by UVC is completely free of pathogens (Faulkner et al., 2024; Snelling et al., 2022).

A second disadvantage, recalling one of in‐duct air filtration, is that in‐duct UVC disinfects pathogens only once they have traveled across the room into the air ducts, potentially infecting occupants along the way. A third disadvantage is that installing in‐duct UVC to an existing HVAC system requires high upfront costs and expertise (Lee & Bahnfleth, 2013; Lee et al., 2009), often higher than upgrading filtration. A fourth disadvantage is that it is not obvious when maintenance is required (Nardell et al., 2013).

#### 3.3.4 Triethylene glycol‐based air disinfectants

The interest in air sterilization by germicidal mists and vapors goes back more than 100 years. Many initial applications used toxic or irritating substances, such as hypochlorite solutions (Trillat, 1938) and smokes (Twort & Baker, 1940). The first use of a glycol (propylene glycol [PG]) to inactivate airborne pathogens appeared in 1941 (Robertson et al., 1941). It was later discovered that TEG is as effective against airborne pathogens (streptococci, pneumococci, and influenza virus) as PG at 250–500 mg/m^3^ but at much lower concentrations (5–10 mg/m^3)^, while being the least toxic of the tested glycols (Robertson et al., 1942, 1943). Further tests demonstrated that TEG was rapidly lethal for hemolytic streptococci, pneumococci, staphylococci, influenza bacilli, the PR8 strain of influenza virus (Puck et al., 1945; Robertson et al., 1943), meningo pneumonitis and psittacosis viruses (Rosebury et al., 1947), airborne mumps virus and Newcastle disease virus (Krugman & Swerdlow, 1949), bacteria including *Bacillus coli* (now recognized as *Escherichia coli* [*E. coli*]) and *subtilis* (vegetative form) (Lester et al., 1952), and a number of common airborne non‐pathogens (Robertson, 1949) and certain molds, like *Penicillium notatum* (Mellody & Bigg, 1946).

Following this success in laboratory studies, TEG was also tested in various hospital settings and barracks to prevent the spread of airborne beta‐hemolytic streptococci, measles, and other airborne pathogens (Hamburger et al., 1945; Puck et al., 1945). While some studies showed significant reductions in airborne pathogens, results on cross‐infections were less clear (Bigg et al., 1945; Krugman & Ward, 1951; Loosli & Smith, 1947).

Due to these insufficiently convincing and limited clinical trials (Bigg et al., 1945; Hamburger et al., 1945; Krugman & Ward, 1951; Loosli & Smith, 1947), overzealous salesmen (Robertson, 1949), and technical problems dispersing TEG and maintaining necessary TEG concentrations, interest in using glycols for air disinfection gradually decreased. Meanwhile, TEG has been used for non‐medical purposes, including creating fog effects in theaters, concerts, and similar venues. These non‐medical uses have led to advances in fog effect technology, and these advances have solved the technical problems of dispersing TEG and maintaining the concentrations necessary for disinfection.

The COVID‐19 pandemic renewed interest in air treatment technologies, and TEG‐based airborne products were suggested to treat the air against infectious organisms. The United States Environmental Protection Agency (EPA) granted an emergency use exemption for a TEG‐based antiviral air treatment, Grignard Pure, for use in certain indoor spaces (US EPA Office of Chemical Safety and Pollution Prevention, 2021). A TEG‐based product, Arresta and a dispersion device are available to purchase and use to eliminate mold odors (Bleu Garde, 2025a, 2025b).

TEG's main *advantages* are high inactivation efficiency against certain airborne agents (Duggan et al., 2024). Airborne MS2 bacteriophage, a commonly used SARS‐CoV‐2 surrogate for experiments, was inactivated with near‐100% efficiency in a matter of minutes in chambers with no ventilation (Desai et al., 2023; Ratliff et al., 2023; US EPA, 2021). This level of deactivation was equivalent to 12.3–28.5 air changes per hour. Under various conditions with ventilation, MS2 has been deactivated at a rate from 8.6 to 16.5 eACH (Sultan et al., 2024). The employed concentrations of TEG were as low as 0.012 mg/m^3^—orders of magnitude lower than those used in studies performed in the 1940s and 1950s (Mellody & Bigg, 1946; Puck, 1947). The mode of action of TEG and other glycols is by dehydration, and since it has been demonstrated to be effective against a wide variety of airborne bacteria and viruses, as mentioned above, it is expected to be effective against viruses with pandemic potential.

Another advantage of TEG is that upfront and ongoing costs are relatively low. A dispenser that can disinfect a space of 600 square feet costs less than $300. One that can disinfect a space of 10,000 square feet costs approximately $1000. Consumption of TEG is 1 mL per 1000 cubic feet per hour (for a room of approximately 11 × 11 × 8). TEG costs approximately $120 per 3785 mL (namely, 1 gallon). This comes to $0.03 per hour and $131.40 per year (assuming 52 weeks of 12 h runtime per day on average) for a 1000 cubic foot space. It follows that disinfecting a 1000 cubic foot space for a year with TEG ($250 for dispenser + $131.40 for TEG liquid) is cheaper than doing so with even the cheapest DIY upper‐room UVC setups ($620) (Davidson, 2021).

A final advantage of TEG is that it is virtually imperceptible. Because the vaporized TEG is usually invisible at typical concentrations, it can be used to provide constant disinfection in most occupied settings; in other cases, it produces only a light haze or fog (Lester et al., 1950). Also, the machines used to disperse the TEG are whisper quiet.

TEG's main *disadvantages* are the need for special equipment to disperse the product, the technological challenge of maintaining the desired levels of TEG in occupied spaces, and people's perceptions and potential concerns that they are surrounded by and breathing a chemical “fog.” Existing data should alleviate the latter concern. Even studies with TEG performed 70–80 years ago emphasized its safety. When TEG vapor was applied in infants’ wards, “there were no complaints from the attendants, nurses, or doctors who worked on the glycol wards. No injurious effect was noted on the respiratory tract or skin of infants who were on the test ward for several weeks to months.” (Loosli & Smith, 1947). In field studies, carried out during cold weather, patients readily adapted themselves to saturated concentrations with no harmful or irritating effects during the limited period of study (Hamburger et al., 1945). Chronic toxicity studies (12–18 months) were carried out on monkeys and rats exposed continuously to air saturated with TEG vapor. No deleterious effects were observed either during life or in histologic sections of the organs following sacrifice at termination of the experiments (Robertson & Loosli, 1947). Thousands of individuals have been exposed to glycol‐containing atmospheres, many of them continuously for months, without apparent disturbance (Robertson, 1949).

Existing animal studies and toxicological reviews show that the product is safe at typical application levels (see Desai et al. [2023] and references therein). The US EPA has concluded that TEG has very low toxicity by the oral, dermal, and inhalation routes of exposure (EPA, 2005), and designated it as a “safer chemical” (PubChem, 2024). Even TEG's cousin, PG, which is needed at higher concentrations to achieve microbial inactivation, is considered “generally regarded as safe” and is approved for wide application for food and drug regulatory agencies (European Medicines Agency, 2014). The latter observation is important because TEG has a lower vapor pressure than PG, and its saturation concentration and, thus, resulting exposures, are lower compared to PG.

Still, tests of TEG in humans are limited. Limited studies reveal associations between high exposure to theatrical glycol‐based fogs and upper respiratory symptoms (Teschke et al., 2005; Varughese et al., 2005), and one recent study detected VOCs released in artificial fog when the glycol fluid was stored in the fog machines for months before use and allowed to oxidize (Guo et al., 2022). These findings raise the possibility that exposure to TEG could cause irritation (e.g., cough, dry throat) among those with respiratory sensitivities or those subject to chronic exposure (see also Guo et al. [2023]).

## 4 INTERIM RECOMMENDATIONS

**FIGURE 1:**
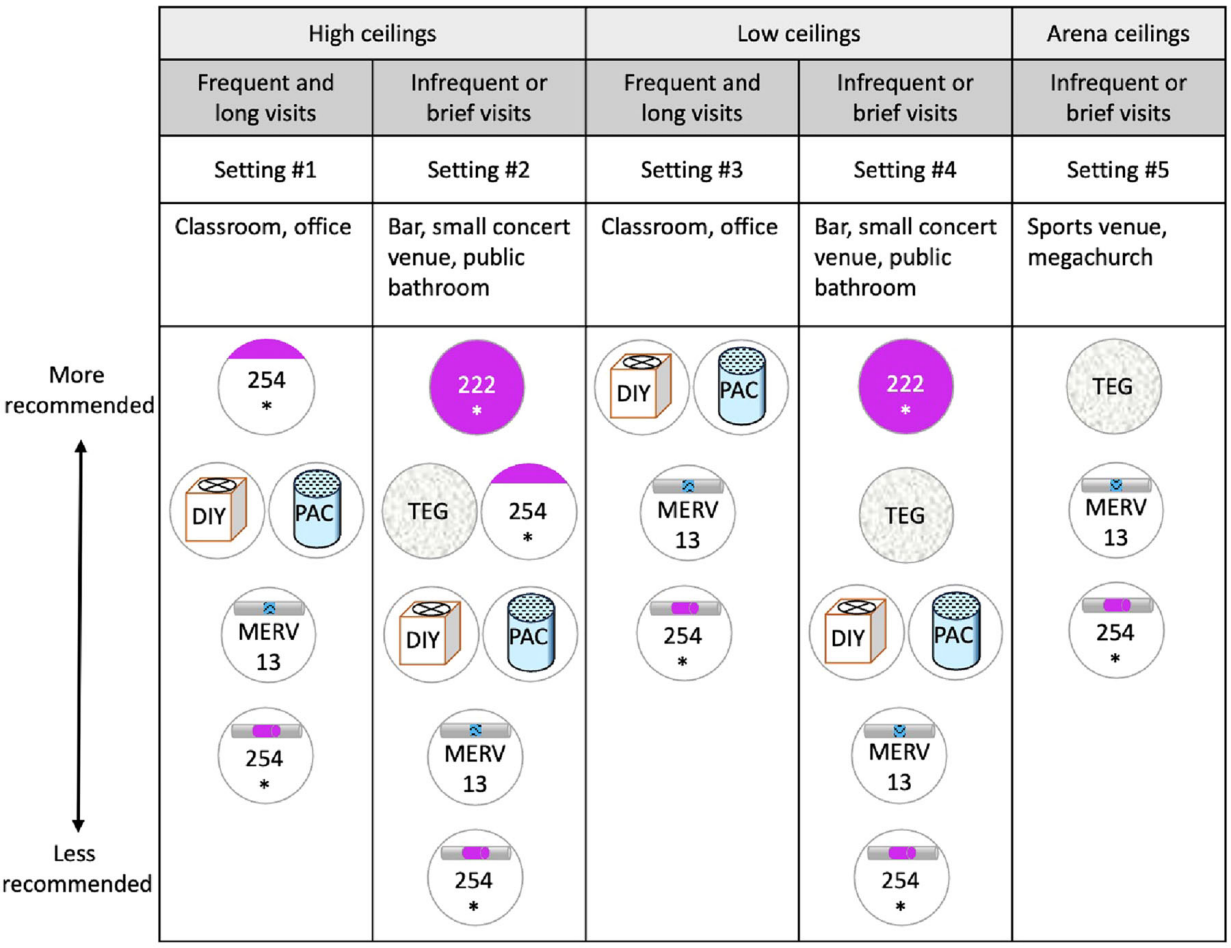
Interim recommendations for deployment. DIY, do‐it‐yourself; MERV, minimum efficiency reporting values; PAC, portable air cleaner; TEG, triethylene glycol.

**FIGURE 2:**
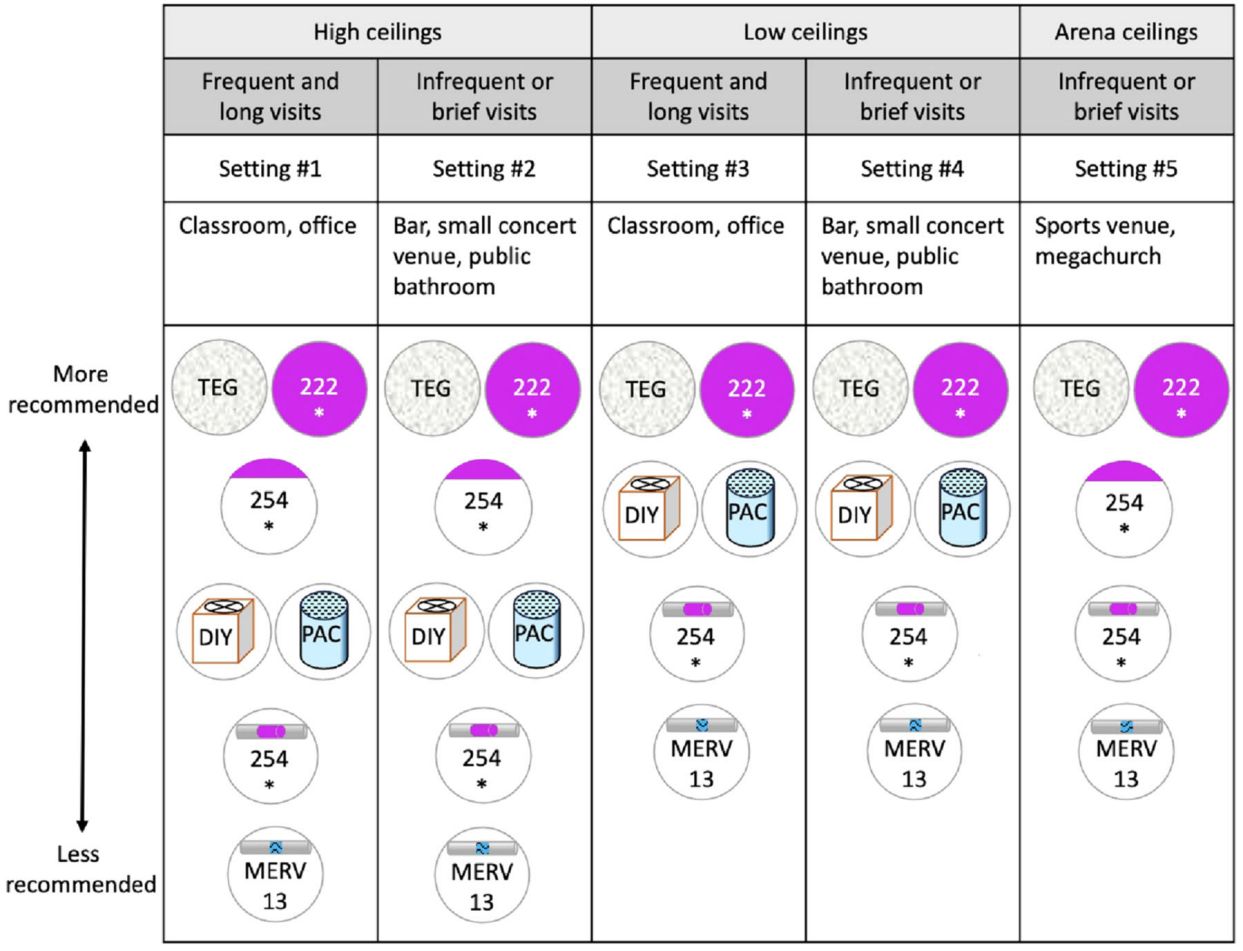
Recommendations for deployment if future research confirms that far‐ultraviolet‐C (UVC) and triethylene glycol (TEG) are safe for long‐term use, effective in real‐world settings, affordable, ethical, and publicly acceptable. DIY, do‐it‐yourself; MERV, minimum efficiency reporting values; PAC, portable air cleaner.

**FIGURE 3:**
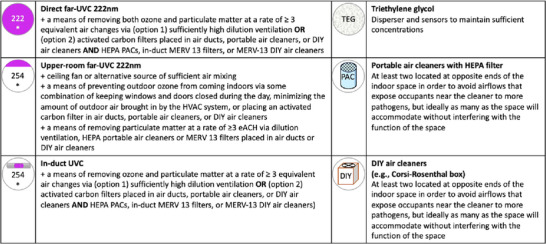
Explanations of the symbols used in Figure 1 and Figure 2. DIY, do‐it‐yourself; HEPA, high efficiency particulate air; HVAC, heating, ventilation, and air conditioning; MERV, minimum efficiency reporting values; PAC, portable air cleaner; TEG, triethylene glycol; UVC, ultraviolet‐C.

Thus far, we have listed the main advantages and disadvantages of different infectious aerosol management strategies. In this section, we recommend which strategies to use in which settings, based on evidence available as of May 2024 (see Figure 1). Importantly, as we emphasize in the next section, these recommendations may change based on the results of future research (see Figure 2 for an example). Figure 3 explains the symbols used in Figures 1 and 2.

We offer these recommendations guided by the goal of maximizing population health. Figure 1 provides a visual summary of these recommendations across different setting types, categorized by ceiling height and occupant exposure patterns. We note, however, that policies that maximize population health may nonetheless be open to legitimate objections. For example, policies that maximize population health might limit individuals’ options to opt out from being subjected to these interventions without limiting access to essential services or participation in society. They may also harm some individuals (say, ones with increased sensitivity to ozone or TEG) or distribute risks, benefits, and burdens unfairly across groups. Although our recommendations often include components intended to avoid these objections, they do not evade all. We provide examples of these objections in Box 3, along with other ethical questions that arise in the course of implementing these recommendations.

Our recommendations differ depending on features of the setting. We divide our recommendations according to ceiling height and the frequency/duration of occupant visits. This is because ceiling height affects whether upper‐room UVC can be utilized safely and effectively, and because the cumulative amount of time an occupant spends in a certain type of setting affects the total level of exposure to the strategy recommended for that setting, and the total level of exposure determines whether they incur any significant long‐term risks associated with that strategy.

To achieve the best protection against highly contagious and/or engineered pathogens, we recommend installing the most effective (and often most expensive) technology, if the costs can be afforded. Otherwise, consider the next best option in our recommended list, which is often more affordable.

### 4.1 Setting #1: High ceilings; potentially high exposure (frequent and lengthy visits)

For indoor air environments with high ceilings and occupants with potentially high exposure (frequent and lengthy visits) (leftmost column of Figure 1), we recommend upper‐room UVC as the primary infectious aerosol management strategy, with three supplements to increase efficacy and safety. First, upper‐room UVC should be deployed alongside air mixing methods, such as ceiling fans (Pichurov et al., 2015) or ventilation (Park et al., 2023), sufficient to move contaminated air from lower‐room to upper‐room, and disinfected air from upper‐room to lower‐room. Second, there must be some means of preventing outdoor ozone from coming indoors, either by keeping windows and doors closed during the day or removing ozone via activated carbon filters in air ducts, PACs, or DIY air cleaners. Third, there must be a method of removing particulate matter at a rate of ≥3 eACH via dilution ventilation, HEPA PACs, or MERV‐13 filters placed in air ducts or DIY air cleaners, without which use of UVC could increase indoor air pollution (Peng, Miller, et al., 2023).

This strategy can secure a rate of disinfection that approaches whole‐room direct far‐UVC and TEG, but avoids lengthy, frequent exposures to the latter two. The recommendation stands until the safety of the latter two is confirmed in population‐based safety studies. While upper‐room UVC can create harmful levels of indoor air pollution, especially when outdoor‐supplied ozone is present, the supplements we recommend substantially minimize that risk. And while improper installation or deliberate misuse of upper‐room UVC can result in skin and eye damage, that damage is only temporary, and with appropriate precautions (e.g., testing dosage levels in occupied portions of the room, installing motion sensors), it rarely arises in practice.

If upper‐room UVC cannot be deployed, we recommend relying on appropriately sized PACs or DIY air cleaners as the primary air management strategy. In particular, we recommend at least two, positioned at opposite ends of the indoor space in order to avoid airflows that expose occupants near each cleaner to more pathogens. In addition, we recommend using as many PACs as the space will accommodate without interfering with the function of the space by generating too much noise or taking up too much floor space. This is in order to maximize CADR, minimize the distance pathogens must travel before being removed by cleaners, and avoid creating airflows that expose occupants near cleaners to a larger total number of pathogen particles. Achieving the same CADR with multiple air cleaners rather than fewer is also advantageous because it produces less noise.

While PACs or DIY air cleaners do not reduce exposure to viable pathogens as quickly and completely as upper‐room UVC, they carry no long‐term health risks. To the contrary, because they also reduce harmful indoor air particulate pollution, they carry long‐term health benefits (Butler & Alper, 2022). On the other hand, the pathogen reduction rate offered by the typical PACs or DIYs will be lower than that provided by upper‐room UVC combined with proper air mixing. (Note: Upper‐room UVC will inactivate the pathogens, but will not physically remove them or their fragments from the air; it is the job of PACs and DIY cleaners to remove inert pathogens.) While the pathogen reduction rate by PACs or DIY air cleaners could be increased by using larger or multiple units, it is not typically feasible to use single PACs or DIYs to achieve the pathogen reduction rate achieved by upper‐room UVC—and using enough air cleaners to do so could be too loud, take up too much space, and either be too expensive (PACs) or too labor‐intensive (DIYs). For that reason, especially against deadly weaponized pathogens, we recommend upper‐room UVC for most spaces along with a small number of PACs or DIYs to remove all particles from the air, including inert pathogens that were inactivated by the upper‐room UVC. This combination minimizes both airborne pathogens and indoor air pollution efficiently, inexpensively, and unobtrusively. Only when upper‐room UVC is not an option do we recommend relying solely on PAC and DIY air cleaners.

The choice between PACs and DIY air cleaners should turn on whether one can afford the more expensive PACs. One should also consider whether the additional expense could fund more cost‐effective DIY air cleaners for many otherwise underprotected spaces and whether it is worth paying more to gain portability, durability, aesthetic appeal, and easier maintenance of PACs.

If PACs and DIY filters cannot be deployed, we recommend upgrading the quality of in‐duct air filtration to MERV‐13, or as high as the HVAC system can handle without an unacceptable pressure drop. Upgrading to MERV‐13 will remove pathogens at a slower rate than our top recommendations. And while it may require a smaller initial investment, it will cause an increase in energy costs that makes it more expensive than either of our top recommendations. We recommend this option only if aesthetic concerns or public mistrust make our top recommendations infeasible.

If the HVAC system cannot provide sufficient airflow through filters with a sufficiently high MERV rating, we recommend in‐duct UVC along with the same supplements we recommend for upper‐air UVC (except for a means of air mixing).

### 4.2 Setting #2: High ceilings; low potential exposure (brief or infrequent visits)

For indoor air environments with high ceilings and occupants with low potential exposure (brief or infrequent visits) (second column from the left in Figure 1), our recommendations are the same as for Setting #1, except that we recommend direct far‐UVC 222 nm as the most recommended strategy, and TEG and upper‐room UVC as second choices. In settings that most users visit only briefly or infrequently, risks of long‐term exposure are low, and so our reasons for ruling out direct far‐UVC and TEG in Setting #1 do not apply.

Considering that TEG is even less expensive than upper‐room UVC and may be more effective as well, there is a plausible case for preferring TEG over upper‐room UVC. However, we do not rank TEG above upper‐room UVC because it has several offsetting disadvantages. First, there are still few studies investigating whether its advantages in efficacy translate to real‐world settings across a variety of pathogen types, whereas upper‐room UVC has a long history of being used in real‐world settings to reduce transmission rates and overall infections. Second, while upper‐room UVC has been introduced in various settings without backlash, there is less evidence that the public would generally accept TEG, even though fog effects created by glycol vapor in entertainment venues face no resistance. Third, although most occupants of Setting #2‐type spaces will only find themselves in those spaces infrequently or briefly, employees in those venues would inevitably be subject to everyday, lengthy exposure to TEG, and so they will incur speculative health risks associated with chronic exposure. Fourth, the widespread use of TEG in most Setting #2‐type spaces frequented by the public, or across other settings, could result in chronic exposure to TEG for a large segment of the population. For example, even though each visit to a bar, public restroom, or public transportation center using TEG will be limited, people may visit several of these sites per day on a daily basis.

Like TEG, direct far‐UVC has the potential to disinfect air even more quickly and completely than upper‐room UVC. However, also like TEG, there is less evidence that direct far‐UVC's high efficacy translates to high effectiveness in real‐world settings, there is less evidence that it will be generally accepted by the public, and employees would inevitably be subject to speculative health risks associated with chronic exposure. Widespread use of direct far‐UVC in Setting #2‐type spaces could also result in chronic exposure. Although direct far‐UVC shares these advantages and disadvantages with TEG, and although we rank TEG on a par with upper‐room UVC, we recommend direct far‐UVC more than both TEG and upper‐room UVC because there is experimental evidence that it can disinfect air far more quickly and completely than both TEG and upper‐room UVC, and the history of upper‐room UVC preventing infection in real‐world settings strongly suggests that direct far‐UVC can do the same. However, direct far‐UVC is also many times more expensive than both TEG and upper‐room UVC. For a limited budget, more spaces can achieve high levels of disinfection, protecting more people, with either TEG or upper‐room UVC than with direct far‐UVC. For this reason, if direct far‐UVC cannot be installed in all managed spaces, we recommend seriously considering TEG and upper‐room UVC.

We recommend both TEG and direct far‐UVC in Setting #2‐type spaces despite the potential health risks of chronic exposure. This is because most occupants of Setting #2‐type spaces will only find themselves in those spaces infrequently or briefly, and, in the case of far‐UVC, we recommend complementing far‐UVC with forms of ventilation and filtration to prevent increased air pollution (Peng, Miller, et al., 2023; Barber 2023). However, employees in those venues would inevitably be subject to everyday, work‐shift, or longer exposure to far‐UVC and TEG, and so they could incur potential health risks, if any, associated with chronic exposure.

Fortunately, studies reviewed above indicate that high levels of disinfection can be achieved with far‐UVC even while ensuring that daily exposure for these employees does not exceed the conservative exposure limits/TLVs set by the ICNIRP or ACGIH before 2022. So risk should be considered acceptable, especially if employees are informed and have adequate opportunity to minimize or avoid exposure altogether. Likewise, as of 2025, the ACGIH has set the TLV‐time‐weighted average (TLV‐TWA) for total inhalable TEG (droplets and vapor) at 10 mg/m^3^ (American Conference of Governmental Industrial Hygienists 2024). Thus, the TEG levels shown to achieve effective inactivation are substantially lower than the current TLV‐TWA. For this reason, potential health risk due to exposure to TEG at typical use concentrations is minimal and should be considered acceptable to employees who are informed of the speculative risks and have adequate opportunity to minimize or avoid such exposure.

In most cases, only employees will be regularly subject to prolonged exposure to far‐UVC and TEG. In rare cases, other regular occupants of an establishment using far‐UVC or TEG (e.g., regular patrons of a neighborhood bar or restaurant) will also be subject to chronic exposures. However, even these exposures will be much shorter and less frequent, falling far below the aforementioned professional exposure limits. In our judgment, the benefits of preventing common infections and engineered pandemics justify using these technologies in individual Setting‐#2 spaces, despite the necessity of further study to rule out speculative risks.

The widespread adoption of far‐UVC and TEG in Setting #2 spaces frequented by the public could lead to chronic exposure for a large segment of the population. However, reaching widespread adoption could take years. In the meantime, we should proceed with deploying TEG and far‐UVC while additional research is conducted to fully understand cumulative risks, if any, associated with frequent, short‐term exposures. Importantly, as the idiom goes, “perception is reality,” so the decision to implement any infectious aerosol management technology must consider likely public reactions to their use. Though the issue of public support for UVC and effective messaging about it is critical to understanding its likely acceptance, there is limited research in the peer‐reviewed literature (Ross et al., 2023) and in online fora (Kraprayoon, 2023) on which to make important decisions. Additional peer‐reviewed research is necessary, ideally using large representative samples of Americans coupled with large samples of those who might be most exposed to UVC. We recommend this research be performed as far‐UVC and TEG are rolled out in a piecemeal fashion.

### 4.3 Settings #3 and #4: Low ceilings

Our rankings for these two settings, where the only difference from Settings #1 and #2 is that ceilings are low (≤9 feet), resemble our rankings for the former two respective settings, except that we rule out upper‐room UVC. When ceilings are high, we usually recommend upper‐room UVC 254 nm (with recommended supplements) as a top choice. But when upper‐room UVC is deployed in spaces with low ceilings, the 254 nm ultraviolet light cannot be restricted to unoccupied portions of the room. There, using upper‐room UVC will consistently produce painful and debilitating effects on occupants unless all occupants wear specialized suits and goggles.

### 4.4 Setting #5: Arena ceilings; low potential exposure (brief or infrequent visits)

Finally, in this setting (arena ceilings, occupants with low potential exposure [infrequent or brief visits]), we recommend TEG, followed by MERV‐13, and then in‐duct UVC. We recommend TEG because it appears to be the only technology that can retain its high rates of disinfection in such a large space without impeding the intended usage of that space. We recommend MERV‐13 and in‐duct UVC as second and third choices because, although they would generate rates of disinfection far lower than TEG, they are, unlike other options, feasible in such a large space without exorbitant expenses.

We rule out PACs and DIY air cleaners because in most cases there would not be enough floor space to deploy the number of cleaners needed to achieve high levels of disinfection. Plus, air mixing due to PACs and DIY air cleaners in cavernous spaces would not be sufficient, creating “short‐circuiting” of airflow around PACs and DIY air cleaners. We rule out far‐UVC both because it would be virtually impossible to bathe the entire space with UVC light without obstructing sight lines or otherwise preventing the intended usage of the space. Moreover, it would be exorbitantly expensive. We rule out upper‐room UVC because it would be virtually impossible to ensure the necessary airflow required to ensure that air passes through the unoccupied space at an appropriate rate. Moreover, it would be expensive and practically impossible to efficiently remove the indoor air pollution generated.

## 5 CONCLUSION AND NEEDED FUTURE RESEARCH

As is evident from the foregoing discussion, there remains a great deal of uncertainty about how different strategies for infectious aerosol management during an engineered pathogen outbreak compare to one another. Many biological, technological, implementational, financial, and ethical questions remain open (see Appendix). This uncertainty makes it difficult to draw definite conclusions about which to pursue. Research that reduces this uncertainty is urgently needed.

We welcome the ongoing studies of efficacy and safety (Derk et al., 2023; Nix et al., 2024; Ratliff et al., 2023; Rockwood, 2021; Zamudio Díaz et al., 2023). Ultimately, however, we need much more research on effectiveness in the field, for example, given real users’ actual levels of trust. Research on how to increase public acceptance of, compliance with, and purchase of the most promising strategies is also needed (see Box 1 for examples). Better numbers on comparative effectiveness could inform research on comparative cost‐effectiveness as well.

Additional research is needed on different combinations of interventions used in particular types of spaces in response to different types of pathogens. For one thing, the incremental benefit of an additional strategy may differ significantly based on which strategies are already in place, the structure of the space, the number of people who occupy that space, how long they occupy that space, what they are doing in that space, and what kind of pathogen one is trying to remove or inactivate (Peng et al., 2022). While it is not feasible to test every possible combination of interventions across all possible settings, we have identified interventions that appear most promising for several common types of high‐risk settings (see Figure 1). These strategy/setting combinations should receive priority in research. Safety and efficacy testing should pertain to their use at the levels required to reduce the spread of highly lethal, highly infectious pathogens (see Box 2 for examples). Finally, we need research and development that expands the range of feasible strategies, investigating novel strategies that may have greater efficacy, greater safety, or lower costs (see Box 3 for examples).

Answering these questions constitutes the first step in analyzing the cost‐effectiveness of the most promising indoor air strategies. But it is not sufficient. This is because, ultimately, effectiveness should not be measured in terms of air changes per hour, CADR, or percent of particles removed, but in terms of health benefits like quality‐adjusted life years or disability‐adjusted life years, or more general measures of well‐being. (Removing 99% of virus particles in a large space with few occupants and near‐universal masking—e.g., a movie theater—may provide much less benefit than removing 95% of virus particles in a small space with many people and imperfect masking—e.g., a subway.) Researchers must synthesize the results of this research to determine how these strategies would affect people's health and well‐being.

Importantly, estimates of an intervention's effectiveness must take into account not only the impacts on the occupants of the space in question, but also downstream impacts on third parties. If possible, studies should also estimate who incurs the costs and who enjoys the benefits of each strategy, allowing decision‐makers to take into account distributive effects and other ethical considerations (See Box 4 for examples). Ethicists must examine whether these ethical considerations undermine the permissibility of the most promising strategies. (See Box 5 for examples.)

Humans spend 90% of their time indoors, and the existing measures for preventing the spread of airborne disease in these spaces are woefully inadequate. If a highly transmissible engineered pathogen were released under the status quo, it would rip through the human population, causing damage orders of magnitude larger than the catastrophe caused by COVID‐19. We can minimize the damage only if we slow the spread of the pathogen soon after it is introduced, and our best hope for doing so is to make our shared spaces inhospitable to airborne pathogens in advance. We offer recommendations about how to do so here, and we believe implementing these recommendations as soon as possible could significantly mitigate the most catastrophic outcomes of an engineered pathogen pandemic while providing significant co‐benefits in the interim. However, if future research confirms that direct far‐UVC and TEG can be safe for long‐term use, effective in real‐world settings, affordable, ethical, and publicly acceptable, our recommendations would shift in favor of these more effective technologies, saving even more lives and potentially avoiding catastrophic outcomes altogether. In any case, answering the research questions we raise here will help us identify how to manage infectious aerosols in the most efficient and ethical way possible.

**BOX 1 Social science research questions**

1. How open are publics to any of these interventions, especially if they require unfamiliar, uncomfortable, energy‐intensive, and possibly permanent behavior changes (e.g., keeping windows open in winter) or active cooperation (e.g., avoiding shared spaces, wearing masks, or even building DIY filters), or sheer passive cooperation (e.g., not interfering with air filtration or UV light systems)?
2. Are there communications and other strategies that we could deploy now to build trust and cooperation with the infectious aerosol management approaches selected?
3. Are some of these approaches more likely to encounter persistent public distrust or non‐compliant behavior than others? 
4. Would public acceptance of these interventions be greater if they were installed and operating at all times versus only after a suspected pandemic outbreak?
5. What incentives and other interventions are likeliest to bring about the desired reform in public, commercial, and private buildings? For example, would it be effective to create an accreditation system like LEED (Leadership in Energy and Environmental Design) for air sanitation, analogous to the Biden administration's recent effort to increase ventilation? (The White House, 2022)
6. How should instructions for building DIY devices be formulated and distributed to maximize build quality and willingness to build DIY devices?

**BOX 2 Medical and engineering research questions**

1. How efficiently does each strategy disinfect air (in m^3^/min) and prevent the spread of the most infectious diseases in Settings #1–#5?
2. How much does each strategy impact indoor air pollution in Settings #1–#5?
3. What are the short‐term health effects of each intervention on occupants of that setting who have skin, eye, or respiratory sensitivities?
4. What are the long‐term health effects of spending typical amounts of time in Settings #1–#5 with each intervention?
5. What is the highest dose of UVC light that can provide the most disinfection without compromising safety?

**BOX 3 R&D questions**

1. Are there innovations that could decrease the price of far‐UVC lighting (e.g., with LEDs [light‐emitting diodes])?
2. Are there innovations that may reduce the risk of accidental exposure to upper‐room UVC at low cost?
3. Are there innovations that may efficiently minimize indoor air pollution generated by UVC‐based interventions at low cost?
4. Are there low‐cost methods the public can use to check the performance of DIY devices?

**BOX 4 Economics research questions**

1. How much does each strategy currently cost?
2. What are the non‐monetary costs and externalities of each strategy?
3. Drawing on answers from earlier research questions, what is the cost‐effectiveness of each strategy in each setting?
4. Who incurs the costs and benefits of each strategy?
5. How can governments use economic policy to lower the costs of the most effective strategies?
6. How can governments create economic incentives that increase the adoption of recommended strategies?

**BOX 5 Ethics research questions**

**Table:** 

Ethical questions	Relevant interventions
1. Paternalism and coercion a. Is it permissible to impose a small risk of harm (e.g., skin cancer, eye damage, respiratory irritation) on an individual without their consent on the grounds that doing so would benefit them (in expectation)? b. If not, does entering a space constitute tacit consent to the risk when the individual has reasonable, less risky alternatives? If so, what constitutes a reasonable, less risky alternative?	Upper‐air UVC Far‐UVC TEG
c. Is it permissible to impose a small risk of harm (e.g., skin cancer, eye damage, respiratory irritation) on an individual without their consent on the grounds that doing so would benefit others (in expectation), if it would also benefit them (in expectation)?	
d. Is it permissible to impose on an individual what he or she incorrectly perceives as a high risk of harm without their consent on the grounds that doing so benefits them?	
2. Aggregate well‐being versus rights against being harmed a. Is an intervention that benefits most people (in expectation) impermissible if, as a side effect, it also foreseeably harms (in expectation) a small group of people (e.g., people with skin or respiratory sensitivities, people who maintain the intervention but forget to turn it off or take precautions) without their consent? b. If so, is it impermissible even if the harmed group has reasonable alternatives to entering spaces whose aerosol management strategies harm them? If that is the case, what constitutes a reasonable alternative?	Upper‐air UVC Far‐UVC In‐duct UVC TEG
3. Responsibility and noncompliance a. If an intervention that benefits most people (in expectation) is impermissible when and because it foreseeably harms a small group (in expectation) as a side effect, does it remain impermissible even if the harmed individuals are harmed only because they put themselves at risk (e.g., by failing or being unable to follow instructions with proper care, taking insufficient precautions, or deliberately misusing technology)? If so, what specific forms of noncompliance would be considered putting oneself at risk?	
b. If an intervention that maximizes population health on the whole disproportionately benefits those who are compliant (e.g., who carefully follow the instructions for building a DIY cleaner) over those who are non‐compliant, is it permissible? Or should we select a policy that fails to maximize population health (e.g., an intervention that is more expensive and therefore saves fewer lives than reliance on DIY cleaners) but distributes health benefits evenly across those who are compliant and those who are non‐compliant? And does the answer depend on *why* the non‐compliant are non‐compliant? c. Is it permissible to choose a policy that fails to maximize population health on the whole if it nevertheless maximizes the health of those who are compliant?	Ventilation PAC DIY filter
4. Doing versus allowing harm a. If the only way to avoid *inflicting* harm on people with an efficacious technology costs additional funds, is it permissible to instead use those funds to buy more of that technology for spaces that are otherwise less protected, *preventing* more harm in aggregate? How does this vary depending on what the decision‐making institution is?	Upper‐air UVC Far‐UVC
5. Fair distribution of benefits a. Under what conditions is it permissible to promote the adoption of technology (e.g., far‐UVC) that can only be used by those who are relatively wealthy or only in select public spaces? How does this vary depending on what institution or actor is doing the promotion, and by what means? b. Should health benefits to some individuals (e.g., essential workers, those who cannot work from home, younger people, older people, worse‐off people, historically marginalized groups) be weighed more heavily than benefits to others, even if protecting those higher priority health benefits requires more expensive technology?	Ventilation Far‐UVC Upper‐air UVC Far‐UVC In‐duct UVC TEG

## 1

**TABLE A1:** Summarizes what is known, and what remains unknown, on the most promising methods for infectious aerosol management. The table is based on mid‐2025 evidence, which is for some squares very limited.

	(a) Equivalent air changes per hour levels^a^	(b) Cost of standard implementation	(c) Health risks	(d) Human challenges to implementation	(e) Technical challenges to implementation	(g) Further investigations necessary
(1) Ventilation using outdoor air	Low (<6)	Low–high	Safe levels of indoor pollutants from both indoor and outdoor origin have yet to be established. In some regions, open windows and doors can introduce noise pollution, air pollution, or insects and other animals carrying disease. Open windows and doors to balconies increase the risk of accidental falls.	Exposure to external elements, noise pollution, air pollution, and temperature while windows are open may discourage compliance. There may be pressure from facilities managers to restrict outside air to low levels to lower heating and/or cooling costs and energy demand. There may be pressure from occupants to restrict outside air to regulate temperature.	Strain on HVAC to adjust environmental parameters of incoming air; periodic replacement of HVAC filters. The building structure has to accommodate the intake of outside air. Requires virtually no expertise and effort to implement but potentially HVAC expertise to maintain.	Options to provide cost‐efficient ways to condition incoming air (e.g., radiative cooling, effective moisture removal). Quantitative estimates of the amount of ventilation required in various source and ventilation supply scenarios and their returns. Effective communications designed to help occupants trust that the level of ventilation with outdoor air is sufficiently protective
(2a) Air filtration: in‐duct	Low (<6)	Moderate–high	Minor risk of exposure to active pathogens when changing filter without sufficient precautions.	Installing systems in new buildings can be cost‐effective, while retrofitting existing systems may be cost‐prohibitive. Because it is not visible, filters may not be replaced with sufficient frequency.	Requires HVAC system Limited impact on large spaces (e.g., large subway stations). Most efficient filters may take up too much space or put too much strain on small systems such as public buses. Requires significant expertise and effort to install but no expertise and minimal effort to maintain. Failure to replace filters can reduce efficacy.	Effective communication strategies designed to help occupants trust that the level of filtration is sufficiently protective and that maintenance routines and recommended schedules for filter changes are being followed.
(2b) Air filtration: Portable air cleaners (PAC)	Moderate (6–12)	Moderate	Minor risk of exposure to active pathogens when changing filter without sufficient precautions.	Noise (60–70 dB) could be disruptive, causing users to lower the fan speed, thus reducing effectiveness. Minor aesthetic concerns may prevent adoption.	Requires floor or wall space. Failure to replace filters can reduce efficacy. Requires moderate expertise to select the appropriate unit for the intended space, little expertise or effort to implement and maintain.	Studies of perception of PAC efficacy and acceptance by people. Studies of effectiveness in real‐world settings. Effective communication strategies designed to help occupants trust that the level of filtration is sufficiently protective and that maintenance routines and recommended schedules for filter changes are being followed.
(2c) Do‐it‐yourself air cleaners	Moderate (6–12)	Low	Minor risk of exposure to active pathogens when changing the filter without sufficient precautions.	Noise (60–70 dB) could be disruptive, causing users to lower the fan speed, thus reducing effectiveness. Major aesthetic concerns may prevent adoption. Because they need to be rebuilt every 6 months, they may not be replaced with sufficient frequency. Some individuals may not feel confident in DIY filters constructed by other non‐experts, inhibiting adoption or willingness to enter public spaces (e.g., classrooms) where DIY filters are relied upon for disinfection. A likely target of accidental damage, but relatively easy and cheap to repair.	Requires floor space. Air leaks (poor build) can reduce efficacy. Requires moderate effort but little expertise to implement and maintain. Somewhat obvious when maintenance is required, but moderate effort required to rebuild can lead to failure to rebuild with sufficient frequency	Studies of public perception and acceptance. Studies of effectiveness in real‐world settings. To the extent that some people will be unwilling to (re‐)build DIY filters with proper care or keep them on high settings, how much will that decrease their cost‐effectiveness? How can governments increase the public's willingness to build DIY cleaners with proper care? If non‐specialists refuse to build DIY cleaners, build only poorly constructed DIY cleaners, or keep them on low settings, are they responsible for any increased risk of infection they incur over the risk they would incur with a well‐constructed DIY cleaner run on high settings?
(3a) Air disinfection: Upper‐room UVC 254 nm light	High–very high (12–‐150)	Moderate	Minor risk of short‐term high‐dose exposure to skin and eyes due to incorrect installation, deliberate misuse, or changing bulbs without sufficient precautions. Negligible risk of long‐term low‐dose exposure to skin and eyes due to incorrect installation. Minor risk from generating indoor air pollution if not paired with suitable ventilation or filtration.	Risks of short‐term exposure and indoor air pollution may inhibit adoption or willingness to enter shared spaces where upper‐room UVC is relied upon for disinfection. A potential target of deliberate damage.	Requires ceilings above 8 feet (ideally above 9 feet). Requires significant expertise and moderate effort to implement but little expertise and effort to maintain. Not obvious when maintenance is required.	Perception and acceptance by people. Studies of effectiveness in real‐world settings What is the property/material damage done to materials that are in the direct light path? Do risks matter less if they fall on those responsible for incorrect precautions or deliberate misuse? Can cheaper, more durable LEDs provide a sufficient source of UVC?
(3b) Air disinfection: In‐duct UVC 254 nm light	Low (<6)	Moderate	Minor risk of short‐term high‐dose exposure to skin and eyes; no risk of long‐term exposure Minor risk from generating indoor air pollution if not paired with suitable ventilation or filtration	Risks of indoor air pollution may inhibit adoption or willingness to enter shared spaces where upper‐room UVC is relied upon for disinfection.	Requires HVAC system Retrofitting existing ductwork may be much more difficult than adding a filter, as it requires a power source at the disinfection point Requires significant expertise and effort to implement and maintain Not obvious when maintenance is required	Studies of public perception and acceptance. Studies of effectiveness in real‐world settings What is the ethics of using this substitute for UVC if this one is less effective? Does it matter ethically if the potential toxicity is avoidable through certain choices? Are some sub‐populations more prone to resulting medical complications (e.g., certain cancers)?
(3c) Air disinfection: Direct far‐UVC 222 nm light	Very high–highest (24–150+)	High	No acute harm to skin or eyes at short exposures. Limited studies with long exposures, but some evidence that the stratum corneum absorbs far‐UVC before it can cause carcinogenic changes in underlying basal cells or melanocytes. Minor risk from generating indoor air pollution if not paired with suitable ventilation or filtration. There is potential for the degradation of plastics and other materials, altering visual appearance and reducing the durability of items in the path of irradiation.	Known risks of short‐term UVB and UVC exposure might lead to (unfounded) perceived risks of short‐term far‐UVC exposure, inhibiting adoption or willingness to enter shared spaces where direct far‐UVC is relied upon for disinfection. Lack of studies on long‐term exposure might lead to perceived risks of long‐term far‐UVC exposure, inhibiting adoption or willingness to enter shared spaces where direct far‐UVC is relied upon for disinfection. A potential target of deliberate damage.	Requires significant expertise and effort to implement, little expertise or effort to maintain. Not obvious when maintenance is required.	Studies of public perception and acceptance. Studies of effectiveness in real‐world settings. Can LEDs provide a cheaper, more durable source? What is the most efficacious dosage that remains safe for long‐term exposure? What are the outcomes of long exposures to skin, eyes, skin microbiome, and pulmonary microbiome? Will assessments that do not rely on UV industry findings reach similarly optimistic conclusions? What is the property/material damage done to materials that are in the direct light path? Is it ethical to promote the adoption of technology that can only be used by those who are relatively wealthy?
(3d) Triethylene glycol (TEG)‐based air disinfectants	High–very high (12–‐150)	Low	Non‐toxic during a long history of use, though with limited intentional testing. No indication of toxicity at typical application levels with proper usage. Unknown risk to individuals with respiratory sensitivities.	Lack of studies on long‐term exposure might lead to perceived risks of long‐term TEG exposure, inhibiting adoption or willingness to enter shared spaces where TEG is relied upon for disinfection. A potential target of deliberate damage because the source can be easily blocked. Generates a “light fog” effect (i.e., reduction in visibility) when used in cavernous spaces, so may inhibit adoption.	Fast acting for initial treatment, but has to be maintained in an indoor space at adequate concentration for continuing effect, especially in the presence of sources. It requires moderate expertise to select appropriate units but little expertise or effort to implement and maintain them. Obvious when maintenance is required.	Studies of public perception and acceptance. Studies of effectiveness in real‐world settings How do focus groups react when it is explained that the fog is not toxic? Do cheaper nudges like a video ad assure the public? Are particular sub‐populations especially anxious about the fog, and how do they respond to communication relaying its safety? What are the ethics of using this intervention if some people will avoid public spaces out of unfounded fear of the fog?

Abbreviations: DIY, do‐it‐yourself; HVAC, heating, ventilation, and air conditioning; UVC, ultraviolet‐C.

^a^ For UVC and TEG, “equivalent air changes” refers to the expected rate at which the intervention would inactivate engineered pathogens. For ventilation and filtration, it refers to the expected rate at which the intervention would remove these pathogens from the air.

**TABLE A2:** Estimates costs to achieve various eACH levels in a 4000 cubic foot room with different infectious aerosol management strategies. Initial costs are for 12‐h daily use, with initial costs amortized over 15 years.

	Range of added eACH per room^a^	15‐year lifecycle cost^b^	$ to achieve ≥24 total eACH in a room	$ to achieve ≥12 total eACH in a room	$ to achieve ≥5 total eACH in a room	$ to add 100 aggregate eACHs	Primary sources of variance in $ per eACH	Strategy description	Sources
Far‐UVC	33–1480	$21,788–$44,542	$0.33–$0.68	$0.33–$0.68	$0.33–$0.68	$0.05–$1.00	Pathogen type, number or power of lamps	4x (15 W, 5000 h) bulbs or 12 (20 W, 10,000 h) bulbs	(Buonanno et al., 2024; Eadie et al., 2022)
Upper‐room UVC	8–150	$10,161–$13,911	$0.15 (when possible)	$0.15 (when possible)	$0.15–$0.21	$0.10–$2.65	Pathogen type, air mixing, humidity	2 × 36″ (40 W, 17,000 h) lamps or equivalent	(Landry et al., 2025; Z. Liu et al., 2024; McDevitt et al., 2008)
In‐duct UVC	4.25–5	$420–$825	–	–	$0.01	$0.13–$0.30	Pathogen type, UVC placement in air handler	Installed in air handler, 85% efficiency, HVAC system achieves 333 CFM in room	(B. Lee et al., 2009)
TEG	8.6–28.6	$668–$905	$2083–$2320	$0.03–$0.04 (when possible)	$0.03–$0.04	$0.11–$0.41	Pathogen type, ventilation rates, model	Bleu Garde Aura or Clearify device + Bleu Garde Arresta	(Desai et al., 2023; Sultan et al., 2024)
PAC	12	$5357–$6991	–	$0.08–$0.11	$0.08–$0.11	$0.68–$0.99	Brand/model	1x (144 W, 800 CFM) PAC or 2x (46 W, 400 CFM) PACs	(Allen & Ibrahim, 2021)
Box fan DIY	12	$4293–$5807	–	$0.07–$0.09	$0.07–$0.09	$0.49–$0.74	Brand/model of fan and filters	1x (166 W) fan with 5 × 2“ MERV‐13 filters or 2x (55 W) fans each with 4 × 1” MERV‐13 filters	(Dal Porto et al., 2022; Derk et al., 2023)
PC fan DIY	12	$3900	–	$0.06	$0.06	$0.49	Brand/model of fan and filters	2x CleanAirKits Luggable XXL (400 CFM each, 13 W	(Intertek, 2023a; Rosenthal, 2023)
MERV‐13 upgrade	3.4–4.25	$1529	–	–	$0.02	$0.55–$0.68	Pre‐existing HVAC fan power and duct design	2 × 1″ MERV‐13 filters replacing filters, clean air flow increased to 333 CFM recirculated air	(Azimi & Stephens, 2013; Bekö et al., 2008; Zaatari et al., 2023)
Outdoor air ventilation increases	5	$220–$4461	–	–	<$0.01–$0.07	$0.07–$1.36	$/kWh and heating/cooling costs due to location	HVAC blowers and ducts can consistently achieve 333 CFM in the room	(Zaatari et al., 2023)

Abbreviations: DIY, do‐it‐yourself; HVAC, heating, ventilation, and air conditioning; MERV, minimum efficiency reporting values; PAC, portable air cleaner; TEG, triethylene glycol; UVC, ultraviolet‐C.

^a^ *Notes on efficacy*: Merv‐13 and in‐duct UVC are expected to have 85% efficiency removing particles between 1 and 3 µm, which includes most infectious aerosols. Lower bound of the eACH range due to any UVC strategy does not include surrogates for notoriously resilient bacteria, such as *Bacillus anthracis*, which are unlikely to trigger a pandemic. Estimates of total eACH assume a baseline of 2.25 ACH. Information was drawn from sources cited in the table, conversations with industry experts, as well as the websites of retailers and manufacturers. Readers with specific questions about calculation methodology and assumptions are welcome to contact the paper's authors for details.

^b^ *Notes on cost*: Ventilation costs are limited to the continental United States, where they are lowest in California and highest in Maine. The cost of electricity for outdoor air ventilation is assumed to vary with the location of the implementation. The cost of electricity for other interventions is assumed to be $0.20 kWh. For all filtration strategies, filters are assumed to be replaced twice yearly. For UVC strategies, bulbs are assumed to be replaced depending on their rated lifespan: 5000‐17,000 h. For TEG, consumption of TEG is assumed to be 1 mL per 1000 cubic feet per hour. The annual cost of calibrating upper‐room UVC is assumed to be $250–$500. The annual cost of other maintenance is assumed to be $50 for upper‐room UVC and $30 for all other strategies. Information was drawn from sources cited in the table, conversations with industry experts, as well as the websites of retailers and manufacturers. Readers with specific questions about calculation methodology and assumptions are welcome to contact the paper's authors for details.
